# Safety and efficacy of bempedoic acid: a systematic review and meta-analysis of randomised controlled trials

**DOI:** 10.1186/s12933-023-02022-z

**Published:** 2023-11-28

**Authors:** Ovidio De Filippo, Fabrizio D’Ascenzo, Mario Iannaccone, Maurizio Bertaina, Attilio Leone, Irene Borzillo, Emanuele Ravetti, Andrea Solano, Ilaria Pagliassotto, Marco Nebiolo, Francesco Bruno, Federico Giacobbe, Saverio Muscoli, Silvia Monticone, Maria Felice Brizzi, Giuseppe Biondi Zoccai, Gaetano Maria De Ferrari

**Affiliations:** 1 Division of Cardiology, Cardiovascular and Thoracic Department, “Città Della Salute e Della Scienza” Hospital, Turin, Italy; 2 Department of Medical Sciences, University of Turin, Corso Bramante 88, 10126 Turin, Italy; 3 Division of Cardiology, San Giovanni Bosco Hospital, ASL Città Di Torino, Turin, Italy; 4 Department of Advanced Biomedical Sciences, University of Naples “Federico II”, Naples, Italy; 5 Division of Cardiology, Fondazione Policlinico “Tor Vergata”, Rome, Italy; 6 Division of Internal Medicine and Hypertension, Department of Medical Sciences, University of Turin, Turin, Italy; 7 Department of Medical-Surgical Sciences and Biotechnologies, Sapienza University of Rome, Latina, Italy

**Keywords:** Bempedoic acid, Meta-analysis, Cardiovascular outcomes, Hypercholesterolemia

## Abstract

**Background and aims:**

Bempedoic Acid (BA) is a novel Lipid-Lowering Therapy (LLT). We performed a systematic review and meta-analysis to assess the efficacy and safety of BA in patients with hypercholesterolemia.

**Methods:**

PubMed, Scopus, and Cochrane library databases were searched for randomised controlled trials evaluating the efficacy and/or safety of BA compared with placebo. Trials investigating dosages other than 180 mg/die were excluded. Major adverse cardiovascular events (MACE) were the primary efficacy endpoint. LDL-cholesterol reduction was the primary laboratory endpoint. Pre-specified safety endpoints included muscle-related adverse events, new-onset diabetes, and gout. The protocol was registered on PROSPERO (temporary ID:399,867).

**Results:**

Study search identified 275 deduplicated results. 11 studies, encompassing 18,315 patients (9854 on BA vs 8461 on placebo/no treatment) were included. BA was associated with a reduced risk of MACE (OR 0.86, 95% CI 0.79–0.95), myocardial infarction (OR 0.76, 95% CI 0.64–0.88) and unstable angina (OR 0.69, 95% CI 0.54–0.88) compared to control, over a median follow up of 87 (15–162) weeks. BA was associated with a reduction of LDL-Cholesterol (mean difference [MD]–22.42,95% CI − 24.02% to − 20.82%), total cholesterol (− 16.50%,95% − 19.21% to − 13.79%), Apo-B lipoprotein (− 19.55%, − 22.68% to − 16.42%) and high-sensitivity CRP (− 27.83%, − 31.71% to − 23.96%) at 12 weeks. BA was associated with a higher risk of gout (OR 1.55, 95% CI 1.27–1.90) as compared with placebo. Efficacy on laboratory endpoints was confirmed, with a variable extent, across patients on statin or ezetimibe background therapy.

**Conclusions:**

The improved cholesterol control achieved with BA translates into a reduced risk of MACE, including myocardial infarction and coronary revascularisation. The drug has a satisfactory safety profile except for an increased risk of gout.

**Supplementary Information:**

The online version contains supplementary material available at 10.1186/s12933-023-02022-z.

## Introduction

Atherosclerotic cardiovascular disease (ASCVD) represents a leading cause of morbidity and mortality. Despite lifestyle interventions and preventive therapies have significantly contributed to reduce its incidence in last decades, 17.8 million people worldwide and almost 4 million people in Europe dye each year because of cardiovascular disease (CVD) [1, 2]. Apolipoprotein B containing (apo-B) lipoproteins, mainly represented by low density lipoprotein (LDL), stand out as a major modifiable risk factors for ASCVD and have been clearly associated with the development of atherosclerotic plaque and major cardiovascular events (MACEs) [3]. Lipid-lowering therapies (LLT) have demonstrated the ability to reduce the incidence of major cardiovascular events [4, 5]. Based on this acknowledged linear relationship between LDL-C level and major cardiac events, there has been a progressive reduction in the international guideline recommended LDL-C targets which are now very ambitious [6].

Statins are the most widely studied and the most prescribed drugs in this setting. However, up to 29% of patients on statins complain about muscle side effects that considerably reduce their therapeutic adherence, particularly when on higher dosages [7]. More recently, proprotein convertase subtilisin/kexin 9 inhibitors (PCSK9-i) have been introduced overcoming these limitations due to a high potency and an extremely favourable side effect profile [5]. However, high cost and associated limited availability of this new class of drugs still reduce the number of candidates for this treatment. As a consequence of these limitations, currently just one out five of the “very high risk” patients reach the LDL-C goal of 55 mg/dl and a very small percentage of patients at “extreme cardiovascular risk” reach the LDL-C goal of 40 mg/dl [8, 9].

Bempedoic Acid (BA) is a new drug that has been recently proposed in this context. It works as an inhibitor of the Adenosine Triphosphate (ATP)-citrate lyase, an enzyme involved in the cholesterol synthesis pathways and acting upstream of (3-hydroxy-3-methylglutaryl coenzyme A (HMG-CoA) reductase) reductase targeted by statins [10]. It has been suggested that the drug-mediated inhibition of ATP-citrate lyase may also restrain vascular smooth muscle cells proliferation and dedifferentiation by activating AMPK/ acetyl-CoA carboxylase signalling pathway, thereby promoting a molecular background to support BA as a therapeutic strategy for diseases associated with intimal hyperplasia such as atherosclerosis [11]. Similarly to statins, BA leads to an upregulation of liver’s LDL receptors and to a reduction of circulating LDL-Cholesterol (LDL-C) levels. Differently from statins, BA is a pro-drug requiring an activating enzyme present in the liver but unexpressed in skeletal muscle (namely the acyl-coenzyme A synthetase-1). Thanks to this characteristic, BA appears to be free of significant muscle side effects. Few controlled studies and meta-analyses have previously demonstrated its ability to reduce cholesterol levels both as single agent and in combination with other LLT, with a good safety profile [12–16].

The larger “Clear Outcome” RCT enrolling nearly 14,000 patients with hypercholesterolemia at high risk of cardiovascular events has just been published and showed a 13% relative risk reduction of MACEs with BA when compared to placebo [17]. Thus, we performed an updated systematic review and meta-analysis of RCTs on BA for hypercholesterolemia treatment. The aims of the analysis were: (1) to provide the most accurate estimate of the effect of BA in reducing the risk of MACE when compared to placebo and the potential interactions with baseline populations’ characteristics; (2) to assess the quantitative reduction of cholesterol levels associated with BA alone or on-top of other LLT (3) to assess its effect on inflammatory markers; (4) to assess its safety and tolerability profile.

## Methods

### Search strategy and selection criteria

In this systematic review and meta-analysis, we searched Pubmed, Scopus and Cochrane library from inception to March 4, 2023. The search strategy for Pubmed is outlined in appendix and included terms as “bempedoic acid, LDL-C, hypercholesterolemia, cholesterol, lipoprotein, low-density lipoprotein, ETC-1002”. Relevant clinical trial registries (Clinical-Trials.gov) were consulted regarding any ongoing studies or the availability of completed studies with reported results. We also checked the reference lists of eligible studies and screened scientific abstracts. We did not use any language or publication status restrictions. To be deemed eligible trials had to meet the following PICOS (patients; intervention, comparison outcomes; study design) criteria: (1) patients with hypercholesterolemia belonging to the following groups: (a) statin intolerant patients or (b) patients on statins with ASCVD, with familiar hypercholesterolemia or with multiple cardiovascular risk factors; (2) intervention: bempedoic acid; (3) comparison: placebo (standard of care or no treatment); (4) at least one clinical (either efficacy or safety) or laboratory endpoint had to be reported; (5) randomised controlled trials. Observational studies, review, case reports, meta-analysis, animal studies and any other studies with a non-randomised design were excluded. Dose-finding studies or studies investigating BA dosages other than 180 mg/die were also excluded as other dosages are not approved for clinical use.

I.P. A.S. and M.N. independently screened the titles and abstracts of retrieved citations to identify relevant studies. All screenings were completed by two researchers independently (I.P., M.N., or A.S.), with disagreements resolved by consensus or with consultation with another author (O.D.F.). I.B. and A.L. reviewed the full-text articles, and any disagreements were resolved by consensus with O.D.F. serving as arbiter. When necessary, corresponding authors of eligible trials were contacted for data verification and missing data in publications, with the aim of gaining additional primary data for meta-analysis.

### Data analysis

Major adverse cardiovascular events (MACE), as defined by included trials, were our primary clinical efficacy outcome, assessed at the latest available follow-up. Secondary efficacy outcome were: all-cause death, cardiovascular (CV) death, myocardial infarction (MI), hospitalization for unstable angina (UA), coronary revascularization and non-fatal stroke. As co-primary efficacy outcome we assessed the relative and absolute reduction of LDL-C at 12 week and at the latest available follow-up. Other secondary outcomes at laboratory level included changes in total cholesterol, HDL-C, non-HDL-C, ApoB lipoprotein and high-sensitivity C reactive protein (hs-CRP) in the BA and in the control groups. As safety outcomes we evaluated new-onset or worsening diabetes, gout, myalgia or muscle disorders, neurocognitive disorders along with any adverse event (AE), serious AE and AE leading to drug discontinuation, and as defined by included trials. Subgroup analyses were performed according to the main inclusion criteria of trials who entered this meta-analysis. In particular, we assessed the efficacy of BA across subgroup of patients with (a) high cardiovascular risk (ASCVD and/or FH and or multiple cardiovascular risk factors); (b) hypercholesterolemic (regardless of medical history of ASCVD and background therapy) and (c) statin intolerant patients. As a sensitivity analysis we excluded from the analysis for the main outcome arms with 100% of patients taking BA and ezetimibe and compared to placebo. Further, we performed several sensitivity analyses to assess the efficacy of BA according to background LLT [namely (a)] high intensity statin vs low/moderate intensity statin vs no statin and (b) ezetimibe vs no ezetimibe). Statin intensity definition was according to current definition of American Heart association guidelines [18]. A metaregression analysis to assess the impact of several baseline variables (Male sex, age, DM and baseline LDL-c) on the efficacy of BA on MACE and LDL-c reduction was also performed.

For studies meeting inclusion criteria, data were independently extracted by three authors (I.B., A.L., E.R.) on standardised templates for outcome measures and study population demographics (population size, age and sex distribution, cardiovascular risk factors, background LLT and baseline metabolic and lipidic profile).

Statistical pooling for incidence estimates was performed with Peto method, computing risk estimates with 95% confidence intervals (CIs) [19]. For continuous variables, statistical pooling was performed according to a random-effect model with generic inverse-variance weighting. Percentage changes from baseline values in BA-treated patients and control group were appraised and expressed as mean difference (MD) and 95% CI. Analyses were performed using RevMan5.2 (The Cochrane Collaboration, The Nordic Cochrane Centre, Copenhagen, Denmark) and Comprehensive meta-analysis (CMA). Hypothesis testing for superiority was set at the two-tailed 0.05 level. Hypothesis testing for statistical homogeneity was set at the two-tailed 0.05 level and based on the Cochran *Q* test, with *I*^*2*^ values of 25%, 50%, and 75% representing mild, moderate, and severe heterogeneity, respectively. Analyses for all outcomes were done on an intention-to-treat basis.

The quality of included studies was independently appraised by two reviewers (M.B. and F.B), with disagreements resolved by consensus. For each RCT, we evaluated the risk of bias (low, moderate, unclear, or high) for randomization, deviation from the intended intervention, missing outcome data, measurement of the outcome and selection of the reported results, in keeping with the Cochrane Collaboration approach [20]. This systematic review and meta-analysis followed PRISMA reporting guidelines and was prospectively registered with the PROSPERO (registration ID:399,867).

## Results

A summary of the screening process and reasons for exclusion is provided in a PRISMA flow diagram (Additional file 1: Fig. S1). A total of 274 records were identified from the electronic databases with an additional 23 records through manually searching journals and clinical trials registries. After removing duplicates, 275 records were screened for the titles and abstracts. Of these, we assessed 20 full-text articles for eligibility and 11 studies were included in the data extraction and quantitative synthesis. The 11 studies globally included 18,315 participants, of whom 10,189 (55.6%) were male and 8126 (44.4%) were female. A summary of features of included studies is reported in Table 1, while full baseline lipid profile and clinical features of patients enrolled are summarized in Additional file 1: Table S1. Briefly, study size ranged from 58 to 13,970 participants. 5 studies enrolled statin-intolerant patients [12, 15, 17, 21, 22], 2 studies enrolled patients with ASCVD and/or FH on stable statin background therapy [13, 14], one multi-arm trial included patients with ASCVD and/or FH and/or multiple CV risk factors [23], 3 trials enrolled patients with hypercholesterolemia [24–26]. Among the latter, two trials enrolled patients on stable or maximally tolerated statin therapy, while the remaining one was a multi arm trial randomising hypercholesterolemic and diabetic patients on wash-out from statins to BA + ezetimibe vs ezetimibe alone vs placebo [26]. For the main analysis the placebo arm served as control, while the ezetimibe arm served as control for the sensitivity analysis investigating the efficacy of BA on top of ezetimibe. 3 studies had a follow up of at least 1 year, whereas the others had follow-up periods less than 1 year (from 4 weeks to 60 months). All trials included used individual patient randomization. The overall risk of bias was low for 7 trials, while 4 trials had an unclear risk of bias in at least one of the explored domains (see Additional file 1: Fig. S2). The definition of main clinical outcomes of each included trial is summarized in Additional file 2: Table S2.

**Table 1:** Main features of included trials

First Author, year (Study acronym)	Country	Study Duration, weeks	Primary outcome	Main Inclusion criteria	Main Exclusion criteria	Background statin therapy	Population	Patients, n		Age	Diabetes (%)	Baseline LDL-C
Ray 2019, (CLEAR HARMONY) [13]	UK	52	Safety (Incidence of adverse events)	Fasting LDL > 70 mg/dl despite maximum tolerated LLT	Use of gemfibrozil or simvastatin at doses greater than 40 mg per day	Maximally tolerated statin therapy	ASCVD and/or FH	BA	1448	65.8 ± 9.1	28.6	103.6 ± 29.1
								Control	742	66.8 ± 8.6	28.6	102.3 ± 30.0
Ballantyne 2019 [21]	US	12	Efficacy (LDL reduction at week 12)	Fasting LDL-cholesterol > 100 mg/dL (ASCVD and/or FH) or > 130 mg/dL (multiple CVD risk factors) despite maximum tolerated statin therapy	Fasting TG ≥ 500 mg/dl BMI ≥ 40 kg/m^2^, recent cardiovascular or cerebrovascular event or procedure	Maximally tolerated statin therapy	ASCVD, and/or FH and/ or multiple CVD risk factors	BA + EZE	108	63.0 ± 10.0	45.4	152.0 ± 39.0
								Control	55	65.6 ± 0.7	43.6	153.0 ± 42.0
Ballantyne 2019 [21]	US	12	Efficacy (LDL reduction at week 12)	Fasting LDL-cholesterol > 100 mg/dL (ASCVD and/or FH) or > 130 mg/dL (multiple CVD risk factors)	Fasting TG ≥ 500 mg/dl, BMI ≥ 40 kg/m^2^, recent cardiovascular or cerebrovascular event or procedure	Maximally tolerated statin therapy	ASCVD, and/or FH and/or multiple CVD risk factors	BA	110	65.2 ± 9.5	56.4	147.0 ± 36.0
								Control	55	65.6 ± 10.7	43.6	153.0 ± 42.0
Goldberg 2019, [12] (CLEAR WISDOM)	US and Europe	52	Efficacy (LDL reduction at week 12)	Fasting LDL > 100 mg/dl despite maximum tolerated LLT	Fasting TG ≥ 500 mg/dL, BMI ≥ 50, eGFR < 30 mL/min/1.73 m^2^, recent CHD event, or clinically significant disease	Maximally tolerated statin therapy	ASCVD and/or FH	BA	522	64.1 ± 8.8	29.7	119.4 ± 37.7
								Control	257	64.7 ± 8.7	31.5%	122.4 ± 38.3
Lalwani 2019 [22]	US	4	Efficacy (LDL reduction at day 29)	Fasting LDL > 100 mg/dl (patients with high-intensity statin therapy), > 115 mg/dl (moderate/low-intensity statin therapy)	Recent or current ASCVD, statin intolerance	High intensity statin therapy	Hypercholesterolemic	BA	41	58 ± 10	NA	71 ± 19
								Control	23	58 ± 8	NA	86 ± 26
Ballantyne 2016 [23]	US	12	Efficacy (LDL reduction at week 12)	Fasting LDL-C levels from 115 to 220 mg/dl and a fasting triglyceride level of 400 mg/dl after washout of lipid-regulating agents	History of clinically ASCVD within 12 months of screening	Maximally tolerated statin therapy	Hypercholesterolemic	BA	45	57 ± 10		142
								Control	45	56 ± 10		131
Laufs 2019 [11] (CLEAR Serenity)	US and Canada	24	Efficacy (LDL reduction at week 12)	Fasting LDL- ≥ 130 mg/dL (primary prevention) or ≥ 100 mg/dL (secondary prevention and/or FH)	Total fasting TG ≥ 500 mg/dL, eGFR < 30 mL/min/1.73 m^2^ BMI ≥ 50 kg/m^2^, recent cardiovascular events or procedure	Lipid lowering agents other than statins and/or very low intensity statin therapy	Statin intolerant patients	BA	234	65.2 ± 9.7	26.9	158.5 ± 40.4
								Control	111	65.1 ± 9.2	23.4%	155.6 ± 38.8
Ballantyne 2018 [14]	US and Canada	12	Efficacy (LDL reduction at week 12)	Statin intolerant patients, requiring additional LDL-C lowering	Clinically significant cardiovascular or cerebrovascular disease; history of coronary or peripheral revascularization	Low intensity or none	Hypercolesterolemic	BA	181	63.7	5	129,8
								Control	88	63.8	6	123
Bays 2021 [24]	US	12	Efficacy (LDL reduction at week 12)	Type 2 diabetes mellitus (HbA1c > 7%) and LDL-cholesterol > 70 mg/dl	BMI > 40 kg/m^2^, documented ASCVD, fasting triglyceride > 400 mg/dL, type 1 diabetes, significant hepatic, renal or hematologic disorder, active malignancy	None (5 weeks washout period)	T2DM and Hypercholesterolemia	BA	60	61.4 ± 9.1	100	145.1 ± 31.5
								Control	119	61.3 ± 8.4	100	141.3 ± 27.2
Rubino 2020 [20]	US	6	Efficacy (LDL reduction at week 6)	Fasting LDL-C 130–189 mg/dL	Cardiovascular disease; (BMI) > 50 kg/m^2^; fasting triglycerides > 400 mg/dL; history of type 1 or type 2 diabetes or f; uncontrolled hypothyroidism; liver, renal or gastrointestinal disorder	None (6 weeks washout period)	Hypercolesterolemic	BA	43	61.2	N/A	154.3
								Control	23	61.2	N/A	155.9
Rubino 2021 [21]	US	8	Efficacy (LDL reduction at 2 month)	Fasting LDL-C levels ≥ 160 mg/dL (without any other lipid lowering agent) and LDL-C levels ≥ 70 mg/dL on PCSK9i	FH, fasting triglyceride levels ≥ 500 mg/dL diabetes, CVD, PAD, uncontrolled hypertension and/or, hypothyroidism, renal, liver, gastroenterological or hematologic disorder	None	Hypercolesterolemic	BA	28	62.0	0	102.1
								Control	30	58.4	0	104.1
Nissen 2023 [16] (CLEAR Outcomes)	32 countries	162,4	Efficacy (MACE incidence)	Statin intolerant patients with increased cardiovascular risk (primary or secondary prevention) with fasting LDL-C levels ≥ 100 mg/dl	Fasting triglycerides ≥ 500 mg/dl, recent acute ASCVD, uncontrolled hypertension, uncontrolled diabetes, hypothyroidism, renal/liver/gastroenterological/hematologic/oncologic disorder	None or very low dose	Statin intolerant patients with ASCVD or at high risk for ASCVD	BA	6992	65.5	45%	139 ± 34.9
								Control	6978	65.5	46.3%	139 ± 35.2

LLT: lipid lowering therapies; ASCVD: atherosclerotic cardiovascular disease; FH: familiar hypercholesterolemia; BA: bempedoic acid; CHD: coronary heart disease; T2DM: type 2 diabetes mellitus; US: united states

**Fig. 1:**
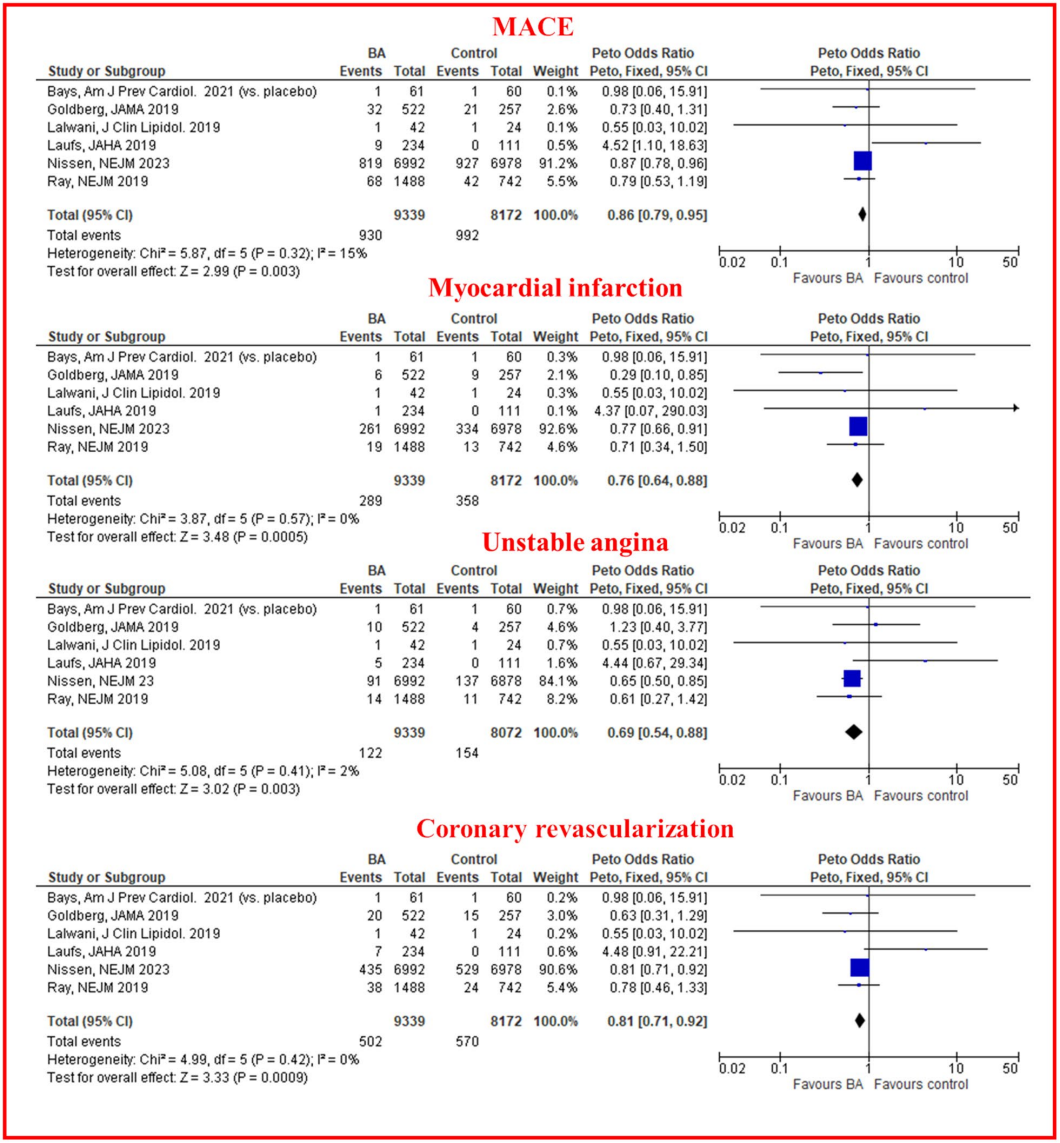
Peto odds ratio for major adverse cardiovascular events (MACE), myocardial infarction, unstable angina and coronary revascularization. BA: bempedoic acid; CI: confidence intervals

Over a median FU of 87 weeks (interquartile range, IQR, 15–162), BA was associated with a 13% reduction of MACE compared with placebo across 6 studies that included 17,511 patients (OR 0.86, 95% CI 0.79–0.95) (Fig. 1). BA was associated with a reduction of MI (OR 0.76, 95% CI 0.64–0.88), unstable angina (OR 0.69, 95% CI 0.54–0.88) and coronary revascularisation (OR 0.81, 95% CI 0.71–0.92), (Fig. 1). No significant difference between BA treated patients and controls was observed with respect to stroke (OR 0.84, 95% CI 0.66–1.06), CV death (OR 1.04, 95% CI 0.88–1.24) and all-cause death (OR 1.05, 95% CI 0.91–1.20), (Additional file 1: Fig. S3). No substantial heterogeneity was observed for any clinical efficacy endpoint. Subgroup analysis according to background medical history for the primary combined endpoint detected a significant reduction of MACE driven from trials including statin intolerant patients treated with BA (OR 0.87, 95% CI 0.79–0.97), whereas the reduction of MACE with BA was not statistically significant different among trials including patients at high cv risk and those enrolling patients with hypercholesterolemia regardless of medical history (see Additional file 1: Fig. S4). Sensitivity analysis for the primary efficacy endpoint, excluding arms testing the combination of BA and Ezetimibe vs placebo confirmed a similar reduction of MACE as compared with the main analysis (see Additional file 1: Fig. S5).

**Fig. 2:**
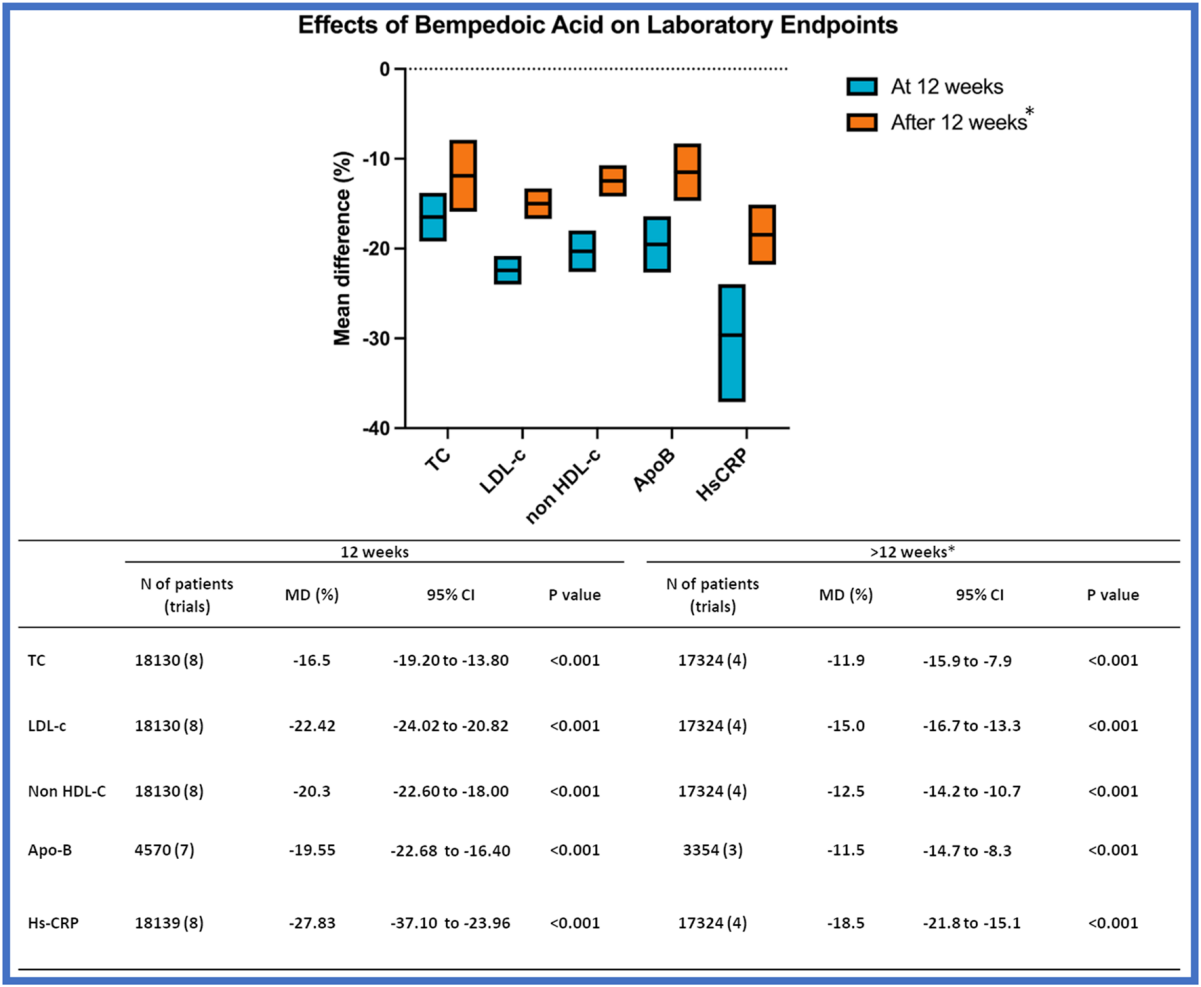
Efficacy of BA on total cholesterol (TC), low density lipoprotein cholesterol (LDL-C), non high-density lipoprotein cholesterol (non HDL-C), Apolipoprotein B (Apo-B) and high sensitivity high reactive protein (hs-CRP). *Median observation period 52 weeks (interquartile range, IQR, 45–79.5) for TC, LDL-C, non HDL-C and hs-CRP); median observation period 52 weeks (IQR 38–52) for ApoB

Effect of BA on secondary laboratory endpoints are summarized in Fig. 2, while full results are displayed in Additional file 1: Fig. S6–8. 8 trials, including 18,130 participants, assessed the change in LDL-C at 12 weeks. Pooled data showed that BA entailed a more significant reduction of LDL-C as compared with control, with a mean difference (MD) of -22.42% (95% CI − 24.0% to − 20.8%). BA was also associated with a significant reduction of TC (MD − 16.5%; 95% CI − 19.2% to − 13.8%), Non-HDL-C (MD − 20.3%; 95% CI 22.6% to − 18.0%), Apo-B lipoprotein (MD − 19–5%; 95% CI − 22.7% to 16.4%) compared with control treatment group at 12 weeks. In parallel, patients on BA experienced a more significant reduction of hs-CPR levels as compared with the control groups (MD − 28.1%; 95% CI − 31.7% to − 24.4%). Significant heterogeneity was observed for all the laboratory efficacy outcomes. Efficacy of BA on lipid profile biomarkers and hs-CPR was consistent at 12 weeks and over a longer observation period (see Fig. 2, Additional file 1: Fig. S7–8). The sensitivity analysis performed to assess the efficacy of BA on % reduction of LDL-c after excluding arms of patients treated with BA and ezetimibe is presented in Additional file 1: Fig. S9 (MD − 19.5%, 95% CI − 20.9% to − 18.2%).

Results of the subgroup analysis assessing the efficacy of BA on laboratory outcomes according to background medical history and trials’ inclusion criteria are summarized in Table 2 (see also Additional file 1: Figs. S10–13).

**Table 2:** Subgroup Analysis according to baseline characteristics of enrolled population for 12 weeks % reduction in laboratory endpoints

	Total cholesterol at 12 weeks			LDL-C reduction at 12 weeks			Non-HDL-c reduction at 12 weeks			Apo B reduction at 12 weeks			Hs-CRP reduction at 12 weeks		
	MD (%)	95% CI	P value	MD (%)	95% CI	P value	MD (%)	95% CI	P value	MD (%)	95% CI	P value	MD (%)	95% CI	P value
High CV risk	− 10.8	− 12.8 to − 8.8	< 0.001	− 17.8	− 18.4 to − 17.2	< 0.001	− 19.0	− 23.3 to − 14.6	< 0.001	− 14.0	− 15.9 to − 12.1	< 0.001	− 19.4	− 30.5 to − 8.3	< 0.001
Hypercholesterolemia	− 19.7	− 34.6 to − 4.8	0.01	− 29.0	− 46.4 to − 11.6	< 0.001	− 24.0	− 41.9 to − 6.0	0.009	− 27.2	− 28.6 to − 25.9	< 0.001	− 39.4	− 41.0 to 37.8	< 0.001
Statin intolerant	− 16.6	− 17.6 to − 15.6	< 0.001	− 22.0	− 24.8 to − 19.2	< 0.001	− 19.8	− 23.3 to − 16.2	< 0.001	− 19.1	− 22.9 to − 15.2	< 0.001	− 29.1	− 34.6 to − 23.6	< 0.001

CV: cardiovascular; MD: mean difference; CI: confidence intervals; Hs-CRP: high sensitivity C reactive protein

Subgroup analysis for percent change in LDL-C from baseline according to statin background therapy is displayed in Additional file 1: Fig. S13. Patients without statin background therapy benefited more on LDL-C lowering at 12 weeks (MD–24.13%, 95% CI − 33.03% to − 15.22%). On top of statins, BA entailed a variable reduction of LDL-c according to statins intensity (MD − 15.67%, 95% CI − 19.05% to − 12.29% and MD − 19.58%, 95% CI − 20.80% to − 18.36% for high, vs moderate/low background statin intensity, respectively). Across 4 studies appraising the incremental efficacy of BA on top ezetimibe background therapy, we found that BA was associated with a mean LDL-c reduction of − 19.03% (95% CI − 22.67 to − 15.39%) when associated to ezetimibe and to a comparable LDL-C reduction without ezetimibe (MD − 18.47–19.80% to − 17.13%), see Additional file 1: Fig. S14.

**Fig. 3:**
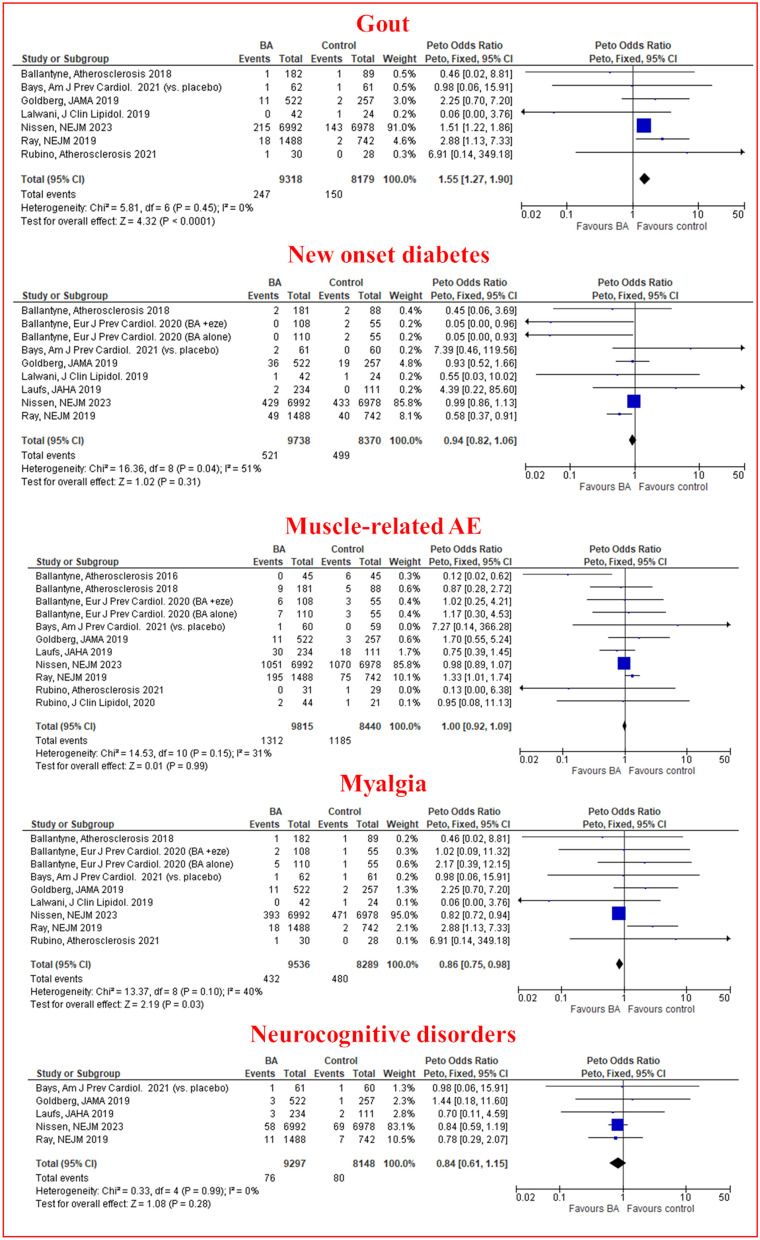
Peto Odds ratio for gout, new-onset diabetes, muscle related adverse events, myalgia, neurocognitive disorders. Legend as in Fig. 1

Pooled results of 7 trials encompassing 17,497 patients indicated that BA was associated with a higher risk of gout as compared with placebo (OR 1.55, 95% CI 1.27–1.90, I^2^ = 0%). On the other hand, BA was not associated with an increased risk of new-onset diabetes (OR 0.94, 95% CI 0.82–1.06, I^2^ = 51%), Fig. 3. As for others safety endpoints no significant differences were observed between BA and control groups for muscle-related adverse events (OR 1.00, 95% CI 0.92–1.09, I^2^ = 31%), myalgia (OR 0.86, 95% CI 0.75-0.98, I^2^ = 40%), and neurocognitive disorders (OR 0.84, 95% CI 0.61–1.15, I^2^ = 0%), Fig. 3. As awaited, a modest increase on the risk of any AE was observed among BA treated patients as compared to placebo (OR 1.13, 95% CI 1.05–1.23, I^2^ = 32%). However, the risk of serious AE (OR 1.03, 95% CI 0.96–1.11, I^2^ = 0%) and discontinuation due to an AE (OR 1.12, 95% CI 0.97–1.30, I^2^ = 29%) was not significantly higher, Additional file 1: Fig. S15.

Results of the metaregression analysis are presented in Additional file 1: Table S3. Increasing age was associated with a less significant reduction of LDL-C between BA-treated patients and controls (Additional file 1: Figs. S16–23).

## Discussion

In this meta-analysis we investigated the safety and efficacy of BA compared to placebo. 11 RCT, encompassing 18,315 patients, were included. Our results can be summarized as follows:

- BA is associated with a substantial and stable in time reduction of LDL-C, total cholesterol, non-HDL-C, Apo-B and hs-CRP as compared to placebo. The efficacy of the drug was confirmed across several subgroups of hypercholesterolemic patients either with ASCVD on maximally tolerated statin therapy or statin intolerant patients.
- BA efficacy on lipid profile translates clinically in a significant reduction of major adverse cardiovascular events, myocardial infarction and the need of coronary revascularisations as compared with placebo or no therapy.
- Efficacy of BA is accompanied by a good safety profile. In particular the incidence of muscle disorders and serious adverse events among patients on BA was comparable to that on placebo. However, a slight but significant higher risk of gout was observed among patients treated with BA as compared to placebo. The recent Clear Outcome study also found a modest increase in cholelithiasis and tendon rupture. No increase in new onset diabetes was observed; rather a trend toward a decrease was found.

To the best of our knowledge this is the largest meta-analysis investigating the clinical properties of BA and including a sample size that is sufficiently powered to assess the efficacy of the drug on hard cardiovascular endpoints following the inclusion of the CLEAR outcome trial [17]. Our findings indeed confirm and extend those of previously published metanalyses focusing on cardiovascular events, where BA proved to be associated with a significant reduction of MACE, myocardial infarction, and myocardial revascularizations, with no substantial signs of heterogeneity or harm [27–29]. While the efficacy of BA in improving the lipidic profile has been previously confirmed both in patients with and without metabolic syndrome [30], the current analysis also allows to assess with greater precision the incremental benefit of the drug across different statin and non-statin background LLT and according to background medical history (either in patients with established ASCD or not). As anticipated, the modulation of the same pathway by both BA and statins results in a decrease in the effectiveness of BA in reducing LDL-C with an increase in the intensity of the associated statin treatment. This difference in efficacy appears modest and possibly lower than anticipated. Indeed, BA provided a reduction of LDL-c ranging from − 15% among patients on high-intensity statins to − 24% among patients not taking statins. On the other hand, the efficacy of BA was constant regardless of ezetimibe background therapy. As ezetimibe is usually well-tolerated and a combination of BA and ezetimibe is available on the market, we may anticipate that this may be offered as a first line therapy in patients requiring 30–40% reduction of LDL-C to achieve their therapeutic goal. Indeed, for these patients, the option of a BA + ezetimibe combination treatment may be a favourable option compared with PCSK-9 inhibitors due to lower costs and easier access. To date only one trial assessed the efficacy of BA on PCKS9-i background therapy [22], reporting a 27.5% incremental reduction of LDL-c over a mean FU of 8 weeks. Altogether, existing evidence suggest that BA may be either used as a single therapy or as a part of a double, triple or quadruple LLT therapy. For instance, across high-risk patients such as diabetics, requiring a LDL-C reduction up to 60%, an upfront triple therapy consisting of statins, BA and ezetimibe could prove beneficial [31].

From a clinical point of view, the drug’s immediate advantage compared to statins is the absence of muscle side effects which make this agent (alone or in combination with ezetimibe) the natural first choice in case of patients unable or unwilling to take statins.

BA use was associated with a significant reduction in unstable angina and myocardial infarction, a trend toward a reduction in stroke but no effect on total and CV mortality. Although we cannot exclude the possibility that this lack of effect may be actually related to the inefficacy of BA on such outcome [32], this finding was not unexpected since a neutral effect on death has been observed in most recent trials of lipid-lowering treatment including studies with shorter follow-up using with more intensive LDL-C lowering [33] and others with longer follow-up and similar LDL-C reduction. It is worth mentioning that recent experimental studies have provided support for the potential use of BA in non-hyperlipidaemic models. These studies have underscored the significance of BA's inhibition of ATP-citrate lyase in macrophages and the liver, which may have implications in preventing the progression of non-alcoholic hepatic steatosis towards fibrosis and in modulating systemic inflammation [34, 35]. These findings suggest that the drug could enhance cardiovascular outcomes through mechanisms that are beyond LDL-C reduction, particularly in peculiar clinical scenarios.

The 14% reduction in MACE observed is in good agreement with the correlation line suggested by the Cholesterol Treatment Trialists Collaboration's meta-analysis [36]. The reduction in MACE found in the most important contributor of this meta-analysis, the CLEAR OUTCOME study is similar to that observed in the composite primary endpoint of the two recent PCSK9i outcome studies [33, 37]. In the latter trials the reduction was achieved in approximately half the follow-up time as compared with CLEAR OUTCOME. Since the cost of treatment with BA is expected to be much less than half that of PCSK9i the pharmacoeconomic profile of BA seems to be favourable, in particular among subjects who may be relatively close to their LDL-C target who may use the BA-ezetimibe combination available at the same cost.

The present data confirm that BA is associated with a significant increase of gout. This effect is attributed to a competition between uric acid and the glucuronide metabolite for the same renal transporter [12] and was suggested to occur mostly in patients with a pre-existing history of gout [17]. Such finding is likely to require clinicians to assess gout risk factors before committing patients to BA, provide dietary guidance, consider alternative antihypertensive medications for at-risk patients currently taking thiazides or loop diuretics, and monitor patients on BA therapy for gout symptoms, promptly initiating management if needed [38]. The modest but significant increases in the incidence of tendon rupture and cholelithiasis observed in the CLEAR outcome [17] trial warrant adequate observational studies to assess their impact on the clinical implementation of BA.

Although this meta-analysis does not confirm the previous suggestion that BA may significantly lower the risk of new-onset DM [16], showing only a numerically non-significant lower incidence of DM, it confirmed that BA does not increase the risk of new onset DM, at variance with statins [39]. The finding that BA acts as an activator of adenosine monophosphate-activated protein kinase (AMPK) [40] increasing insulin sensitivity and improving glycemic control in the liver [41] may be the underlying mechanism of this effect. Similarly to statins, but differently from other non-statins agents such as PCSK9i, BA significantly reduced the inflammatory marker CRP. Whether this activity contributes to the overall benefit of the agent is unknown, but it is acknowledged that an anti-inflammatory activity may reduce MACE in patients with coronary artery disease.

Our results must be interpreted in the context of some limitations. Like all meta-analyses, this study shares the limitations of each individual trial included. Nevertheless, the trials analysed in this study were all randomized controlled trials with an estimated low or moderate risk of bias. We did not have access to individual-level data, so we were unable to assess the potential effect of patient heterogeneity. As some studies were phase II trials, there exists heterogeneity in terms of trial design, sample size, power, and follow-up.

Notably, the most recent trial accounts for over 70% of the entire sample in this meta-analysis, potentially explaining the low heterogeneity for most of the analysis on the clinical events, along with similar inclusion criteria and consistency of results. Such trial was also characterised by a considerably longer follow-up as compared with other included trials. To account for the potential bias associated with this issue, analyses were performed at 12-weeks and at the latest available follow-up whenever feasible.

Despite these limitations, our results provide comprehensive and up-to-date evidence about the safety and efficacy of BA, including its impact on lipid profiles and cardiovascular outcomes. Additionally, for the first time, we explored the potential of BA when used in conjunction with other background LLTs trying to better quantify the relative effect of this new drug. Indeed, the present paper compared to other meta-analysis on the same topic enrolled a larger sample size and provided insights of the effect of BE on clinical events and on lipid profile controls of patients stratified according to indications to addictive therapy (that is statin intolerant vs. high CV risk vs hypercholesterolemia) and on lipid profile according to background medical therapy.

In conclusion, our meta-analysis provides comprehensive and updated evidence supporting the efficacy of BA in reducing LDL-C, total cholesterol, Apo B, and hs-CRP, resulting in a significant reduction of MACE including myocardial infarction. Use of BA is associated with a satisfying safety profile.

Also taking into consideration the possible combination with ezetimibe, our findings suggest that BA may represent a useful therapeutic option for patients with dyslipidaemia and high cardiovascular risk, especially in those unable or unwilling to take statins.

### Supplementary Information

**Additional file 1: Figure S1.** PRISMA flowchart for study selection. **Table S1.** Baseline features of included patients. **Figure S2**. Risk of Bias assessment of included trials. **Figure S3.** Peto Odds ratio for stroke, cardiovascular death and all-cause death.. **Figure S4**. Risk of MACE according to inclusion criteria and background medical history. (excel sheet provided separately). **Figure S5.** Sensitivity analysis for MACE (excluding trials with arms of BA and ezetimibe). **Figure S6.** Efficacy of BA compared to control for percentage reduction at 12 weeks of LDL-cholesterol, total cholesterol and non-HDL cholesterol, apolipoprotein B, high-sensitivity C reactive protein (hs-CRP). **Figure S7.** Efficacy of BA on laboratory endpoints at latest available follow-up: effect of LDL-C, total cholesterol, and non-HDL-C. **Figure S8.** Efficacy of BA on laboratory endpoints at latest available follow-up: effect on Apo-B and hs-PCR. **Figure S9.** Sensitivity analysis: efficacy of BA on % reduction of LDL-c after excluding arms of BA + ezetimibe. **Figure S10**. Efficacy of BA on LDL-C % reduction according to background medical history and trials’ inclusion criteria. **Figure S11.** Efficacy of BA on total cholesterol and non HDL-cholesterol according to background medical history and trials’ inclusion criteria. **Figure S12.** efficacy of BA on HS CPR and ApoB lipoprotein according to background medical history and trials’ inclusion criteria. **Figure S13.** Efficacy of BA on LDL reduction according to statin background therapy. **Figure S14.** Efficacy of BA on LDL reduction according to background ezetimibe therapy. **Figure S15.** Risk of any adverse event, serious adverse events and drug discontinuation due to an adverse event. **Table S3.** Metaregression analysis. **Figure S16.** Metaregression analysis. Impact of age on the risk of MACE. **Figure S17.** Metaregression analysis. Impact of male gender on the risk of MACE**. Figure S18.** Metaregression analysis. Impact of baseline LDL-C on the risk of MACE. **Figure S19.** Metaregression analysis. Impact of diabetes on the risk of MACE. **Figure S20**. Metaregression analysis. Impact of age on the difference in reduction of LDL-c between patients receiving bempedoic acid and control treatment group. **Figure S21.** Metaregression analysis. Impact of male gender on the difference in reduction of LDL-c between patients receiving bempedoic acid and control treatment group. **Figure S22.** Metaregression analysis. Impact of baseline LDL-c on the difference in reduction of LDL-c between patients receiving bempedoic acid and control treatment group. **Figure S23.** Metaregression analysis. Impact of diabetes on the difference in reduction of LDL-c between patients receiving bempedoic acid and control treatment group.

**Additional file 2: Table S2.** Endpoint definition. MACE: major adverse cardiovascular events; AE: adverse event, TEAE: treatment-emergent adverse event, AESI: adverse events of special interest.

## Data Availability

As a study-level meta-analysis, no new data were generated or analysed in support of this research. Derived data used to calculate meta-estimates will be shared on reasonable request to the corresponding author.
