# Supplementary material for: Safety and efficacy of bempedoic acid: a systematic review and meta-analysis of randomised controlled trials
Source: Cardiovasc Diabetol. 2023 Nov 28;22:324. doi: 10.1186/s12933-023-02022-z (PMC10685600; doi:10.1186/s12933-023-02022-z)

**Safety and efficacy of Bempedoic Acid: a systematic review and metanalysis of randomized controlled trials.**

Additional file appendix

Table of contents

**Search strategy** (PubMed example)

**Additional file 1: Figure S1.** PRISMA flowchart for study selection

**Additional file 1: Table S1.** Baseline features of included patients.

**Additional file 1: Figure S2**. Risk of Bias assessment of included trials.

**Additional file 1: Figure S3.** Peto Odds ratio for stroke, cardiovascular death and all-cause death.

**Additional file 1: Figure S4**. Risk of MACE according to inclusion criteria and background medical history. (excel sheet provided separately)

**Table S2.** Endpoint definition (provided in a separate file)

**Additional file 1: Figure S5.** Sensitivity analysis for MACE (excluding trials with arms of BA and ezetimibe)

**Additional file 1: Figure S6.** Efficacy of BA compared to control for percentage reduction at 12 weeks of LDL-cholesterol, total cholesterol and non-HDL cholesterol, apolipoprotein B, high-sensitivity C reactive protein (hs-CRP).

**Additional file 1: Figure S7.** Efficacy of BA on laboratory endpoints at latest available follow-up: effect of LDL-C, total cholesterol, and non-HDL-C

**Additional file 1: Figure S8.** Efficacy of BA on laboratory endpoints at latest available follow-up: effect on Apo-B and hs-PCR

**Additional file 1: Figure S9.** Sensitivity analysis: efficacy of BA on % reduction of LDL-c after excluding arms of BA+ ezetimibe.

**Additional file 1: Figure S10**. Efficacy of BA on LDL-C % reduction according to background medical history and trials’ inclusion criteria

**Additional file 1: Figure S11.** Efficacy of BA on total cholesterol and non HDL-cholesterol according to background medical history and trials’ inclusion criteria

**Additional file 1: Figure S12.** efficacy of BA on HS CPR and ApoB lipoprotein according to background medical history and trials’ inclusion criteria

**Additional file 1: Figure S13.** Efficacy of BA on LDL reduction according to statin background therapy.

**Additional file 1: Figure S14.** Efficacy of BA on LDL reduction according to background ezetimibe therapy

**Additional file 1: Figure S15.** Risk of any adverse event, serious adverse events and drug discontinuation due to an adverse event

**Additional file 1: Table S3.** Metaregression analysis

**Additional file 1: Figure S16.** Metaregression analysis. Impact of age on the risk of MACE

**Additional file 1: Figure S17.** Metaregression analysis. Impact of male gender on the risk of MACE

**Additional file 1: Figure S18.** Metaregression analysis. Impact of baseline LDL-C on the risk of MACE

**Additional file 1: Figure S19.** Metaregression analysis. Impact of diabetes on the risk of MACE

**Additional file 1: Figure S20**. Metaregression analysis. Impact of age on the difference in reduction of LDL-c between patients receiving bempedoic acid and control treatment group

**Additional file 1: Figure S21.** Metaregression analysis. Impact of male gender on the difference in reduction of LDL-c between patients receiving bempedoic acid and control treatment group

**Additional file 1: Figure S22.** Metaregression analysis. Impact of baseline LDL-c on the difference in reduction of LDL-c between patients receiving bempedoic acid and control treatment group

**Additional file 1: Figure S23.** Metaregression analysis. Impact of diabetes on the difference in reduction of LDL-c between patients receiving bempedoic acid and control treatment group

**Search strategy**

**PubMed search Keywords Number of results**

(bempedoic acid OR ETC-1002) 60

(bempedoic acid OR ETC-1002) AND (cholesterol) 228

(bempedoic acid OR ETC-1002) AND (cholesterol OR hypercholesterolemia) 245

(bempedoic acid OR ETC-1002) AND (cholesterol OR hypercholesterolemia OR hypercholesterolemic) 245

(bempedoic acid OR ETC-1002) AND (cholesterol OR hypercholesterolemia OR hypercholesterolemic OR lipoprotein) 247

(bempedoic acid OR ETC-1002) AND (cholesterol OR hypercholesterolemia OR hypercholesterolemic OR lipoprotein OR LDL) 248


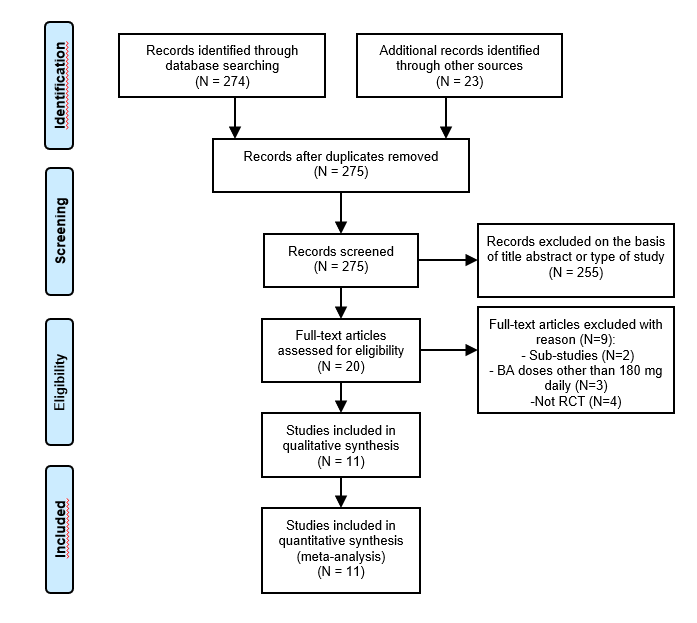


**Additional file 1: Figure S1.** PRISMA flowchart for study selection

**Additional file 1: Table S1.** Baseline features of patients enrolled in trials included in the meta-analysis. BMI: body mass index; HDL-C: high density lipoprotein cholesterol; LDL-C: low density lipoprotein cholesterol; APO-B: apolipoprotein-B; Hs-CPR: high sensitivity C-reactive protein; T2DM: type 2 diabetes mellitus

| **First Author, year**  **(Study acronym)** | **Population** | | | **Patients, n** | **Male (%)** | **Ezetimibe (%)** | **Statin (%)** | **High intensity statin (%)** | **BMI** | **ASCVD (%)** | **FH (%)** | **Hypertension** | **Diabetes (%)** | **Baseline total Cholesterol** | **Baseline HDL-C** | **Baseline**  **Non HDL-C** | **Baseline LDL-C** | **Baseline triglycerides** | **Apo-B** | **Baseline Hs- CPR** |
| --- | --- | --- | --- | --- | --- | --- | --- | --- | --- | --- | --- | --- | --- | --- | --- | --- | --- | --- | --- | --- |
| Ray 2019,  (CLEAR HARMONY) | ASCVD and/or FH | | BA | 1448 | 73.9 | 7.8 | 99.8 | 49.9 | N/A | 97.4 | 3.8 | 78.9 | 28.6 | 179.7 ± 35.1 | 48.7 ± 11.9 | 130.9 ± 33.7 | 103.6 ± 29.1 | 126  (98-166) | 88.5 ±21.6 | 1.49  (0.74- 3.28) |
|  |  |  | Control | 742 | 71.3 | 7.5 | 100 | 49.9 | N/A | 98 | 3.1 | 80.1 | 28.6 | 178.6 ± 35.6 | 49.3 ± 11.5 | 129.4 ± 33.9 | 102.3 ± 30.0 | 123  (96-170) | 86.8 ±21.8 | 1.51  (0.79-3.3) |
| Ballantyne 2019 | CAD and/or FH and/or multiple CV risk factors | | BA + EZE | 108 | 46.3 | 100 | 69,4 | 38,9 | 31,2 | 55,6 |  | 87 | 45.4 | 236.0 ± 49.0 | 49.0 ± 15.0 | 188.0 ± 47.0 | 152.0 ± 41.0 | 157  (106-209) | 121.1 ± 30.9 | 3.1  (1.7-6.2) |
|  |  |  | Control | 55 | 60 | 0 | 74,5 | 21 | 30,5 | 56,4 |  | 85,5 | 43.6 | 233.0 ± 50.0 | 50.0 ±14.0 | 181.0 ± 50.0 | 153.0 ± 47.0 | 139  (105-168) | 115.1 ± 32.5 | 3  (1.4-5.5) |
| Ballantyne 2019 | CAD and/or FH and/or multiple CV risk factors | | BA | 110 | 40,9 | 0 | 75,4 | 36,4 | 30,6 | 57,3 |  | 89,1 | 56.5 | 229.0 ± 43.0 | 50.0 ± 12.0 | 176.0 ± 41.0 | 147.0 ± 41.0 | 141  (108-190) | 113.4 ±26.4 | 2.9  (1.4-5) |
|  |  |  | Control | 55 | 60 | 0 | 74,5 | 21 | 30,5 | 56,4 |  | 85,5 | 43.6 | 233.0 ± 50.0 | 50.0 ± 14.0 | 181.0 ± 50.0 | 153.0 ± 47.0 | 139  (105-168) | 115.1 ± 32.5 | 3  (1.3-5.5) |
| Goldberg 2019,  (CLEAR WISDOM) | ASCVD and/or FH | | BA | 522 | 62.8 | 7.3 | 90 | 53.3 | 30 | 94.8 | 5.2 | 83.9 | 29.7 | 202.1 ± 42.7 | 51.4 ± 12.9 | 150.7 ± 42.7 | 119.4 ± 37.7 | 139  (102-190) | 116.2 ± 29.6 | 1.61  (0.87-3.46) |
|  |  |  | Control | 257 | 65.4 | 9.3 | 88.8 | 52.5 | 30.6 | 93.8 | 6.2 | 87.2 | 31.5 | 204.8 ± 46.1 | 51.1 ± 13.1 | 153.7 ± 44.4 | 122.4 ± 38.3 | 143  (106-189) | 118.6 ± 30.5 | 1.88  (0.92-3,79) |
| Lalwani 2019 | Hypercholesterolemic | | BA | 41 | 48,8 | 0 | 100 | 100 | 31 | 0 | 0 | NA | NA | 146.0 ± 27.0 | 49.0 ± 16.0 | 96.0 ± 24.0 | 71.0 ± 19.0 | 104  (52-331) | 70 ± 15 | 3.2  (0.1-14.8) |
|  |  |  | Control | 23 | 56,5 | 0 | 100 | 100 | 31 | 0 | 0 | NA | NA | 161.0 ± 28.0 | 47.0 ± 9.0 | 114.0 ± 28.0 | 86.0 ± 26.0 | 124  (74-286) | 82 ± 21 | 2.5  (0.1-17.0) |
| Ballantyne 2016 | Hypercholesterolemic | | BA | 45 | 52 | 0 | 100 | NA | 30 | 0 | NA | NA | NA | 229.0 ± 29.0 | 55.0 ± 14.0 | NA | 142.0 ± 28.0 | 145  (122-196) | NA | 1.8  (1.20-4.00) |
|  |  |  | Control | 45 | 32 | 0 | 100 | NA | 31 | 0 | NA | NA | NA | 212.0 ± 24.0 | 54.0 ± 14.0 | NA | 131.0 ± 22.0 | 119  (82-159) | NA | 1.8  (1.10-4.60) |
| Laufs 2019 | Statin intolerant patients | | BA | 234 | 43.2 |  | 18 | 0 | 30.1 | 38.5 | 1.7 | 67.5 | 26.9 | 245.7 ± 47.3 | 52.2 ± 14.5 | 193.5 ± 45.1 | 158.5 ± 40.4 | 156.5  (114.5-219.0) | 141.0 ±31.6 | 2.92  (1.34, 5.29) |
|  |  |  | Control | 111 | 45 |  | 11 | 0 | 30.6 | 39.6 | 2.7 | 67.6 | 23.4 | 241.1 ± 44.3 | 50.4 ± 14.4 | 190.7 | 155.6 ± 38.8 | 164.0  (120-225.5) | 141.9 ± 30.4 | 2.78  (1.21, 5.15) |
| Ballantyne 2018 | Hypercolesterolemic | | BA | 181 | 39.8 | 100 | 21 | 0 | 29.5 | 27.1 | NA | 61.3 | 19.3 | 228.2 ± 35.9 | 55.8 ± 16.3 | 162.4 ± 35.4 | 129.8 ± 30.9 | 153.0  (12.0-209.0) | 123.33 ± 26.5 | 2.21  (1.10, 4.00) |
|  |  |  | Control | 88 | 36.7 | 100 | 8 | 0 | 30.5 | 25.0 | NA | 58.0 | 19.3 | 206.8 ± 35.7 | 57.1 ± 21.3 | 151.7 ± 32.7 | 123.0 ± 27.2 | 135.5  (99.8-175.8) | 115.88 ± 23.5 | 2.26  (1.06, 4.50) |
| Rubino 2020 | Hypercolesterolemic | | BA | 43 | 40 | 5 | 56 | N/A | 29.3 | 0 | 29,3 | 53 | 0 | 235.7 ± 27.9 | 51.2 ± 12.9 | 183.1 ± 18.4 | 154.3 | 126.5  (88.0-190.5) | 118.0 ± 19.9 | 1.0  (1.04-7.0). |
|  |  |  | Control | 20 | 30 | 5 | 45 | N/A | 28.4 | 0 | 2.44 | 35 | 0 | 235.1 ± 21.2 | 52.0 ± 13.0 | 183.1 | 155.9 | 125.0  (90.2, 171.0) | 119.2 ± 15.2 | 1.6  (0.9, 3.2) |
| Bays 2021 | T2DM and Hypercholesterolemic | | BA | 60 | 55 | 100 | 0 | 0 | 31.1 | 0 | 0 | NA | 100 | 230.3 ± 37.2 | 48.7 ± 13.1 | 181.7 ± 36.7 | 145.1 ± 31.5 | 172.2  (128-239) | 121.6 ± 23 | 2.6  (1.7-4.9) |
|  |  |  | Control | 119 | 50.5 | 50 | 0 | 0 | 31.4 | 0 | 0 | NA | 100 | 223.6 ± 33.2 | 48.2 ± 11.7 | 175,1 ± 31.2 | 141.3 ± 27.3 | 161  (118-236) | 119 ± 20.7 | 2.9  (1.5-7) |
| Rubino 2021 | Hypercolesteolemic | | BA | 28 | 25 | 0 | 0 | 0 | 2.,6 | 0 | 0 | NA | 0 | 186.2 ± 38.3 | 56.5 ± 12.3 | 129.7 ± 36.3 | 102.1 ± 29.0 | 130.2  (92.8-176,5 | 87.5 ± 27 | 3.1  (1.3-4.6) |
|  |  |  | Control | 30 | 50 | 0 | 0 | 0 | 28.2 | 0 | 0 | NA | 0 | 190.3 ± 39.4 | 57.3 ± 15.5 | 133.0 ± 36.6 | 104.1 ± 32.1 | 129.0  (101-190.5) | 87.9 ± 28.3 | 2.1  (1.1, 4.7) |
| Niessen 2023 | | Statin intolerant patients | BA | 7001 | 51.8 | 11.5 | 22.9 | N/A | 29.9 | 70 | N/A | N/A | 45.6 | 223.5±40.6 | 49.6±13.3 | 173.8±39.5 | 139.0±34.9 | 159.5 (118.0–216.5) | N/A | 2.3  (2.0-4.5) |
|  |  |  | Control | 6964 | 51.9 | 11.6 | 22.5 | N/A | 30.0 | 69.8 | N/A | N/A | 45.9 | 223.3±41.1 | 49.4±13.3 | 173.9±40.2 | 139.0±35.2 | 158.5 (118.0–215.0) | N/A | 2.3  (2.0-4.5) |

**Additional file 1: Figure S2.** Risk of Bias assessment of included trials.

**
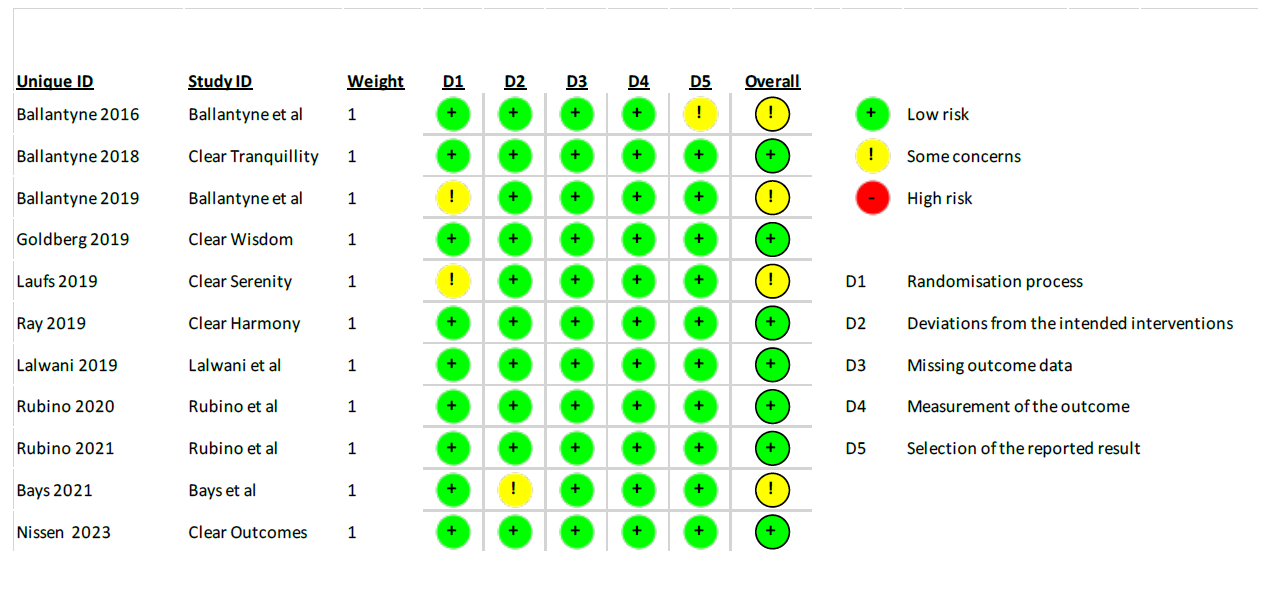
**

**Additional file 1: Figure S3.** Peto Odds ratio for stroke, cardiovascular death and all-cause death.


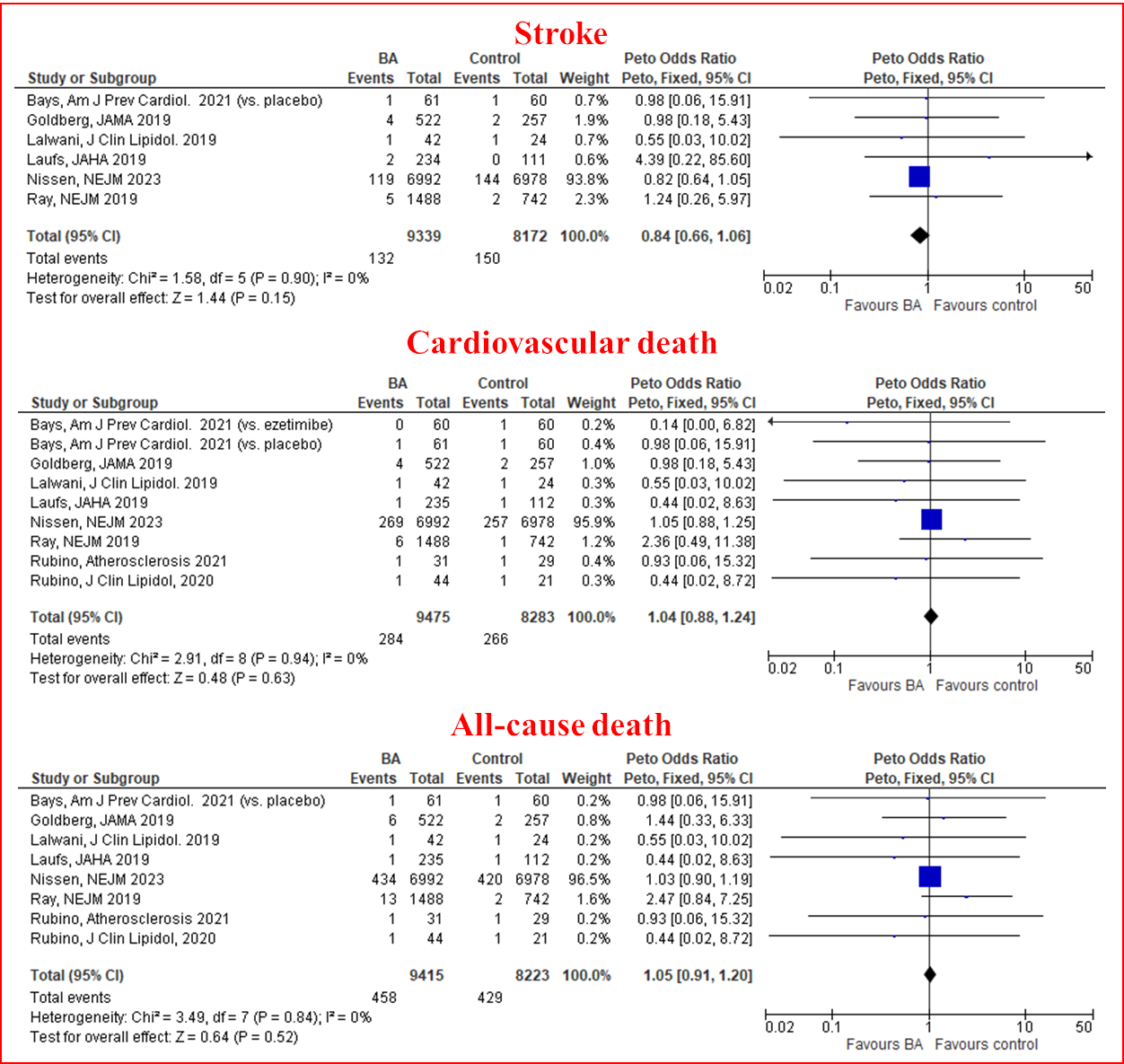


Legend as in figure 1.

**Additional file 1: Figure S4.** Risk of MACE according to inclusion criteria and background medical history.


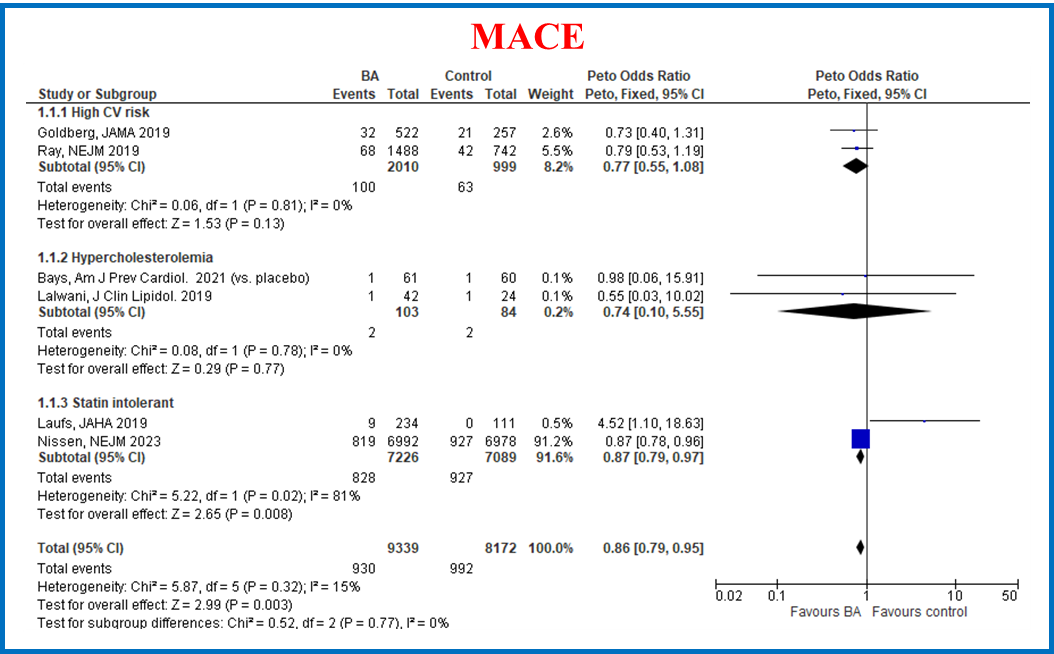


Legend. CI: confidence intervals; BA: bempedoic acid.

**Additional file 1: Figure S5.** Sensitivity analysis for MACE (excluding trials with arms of BA and ezetimibe)


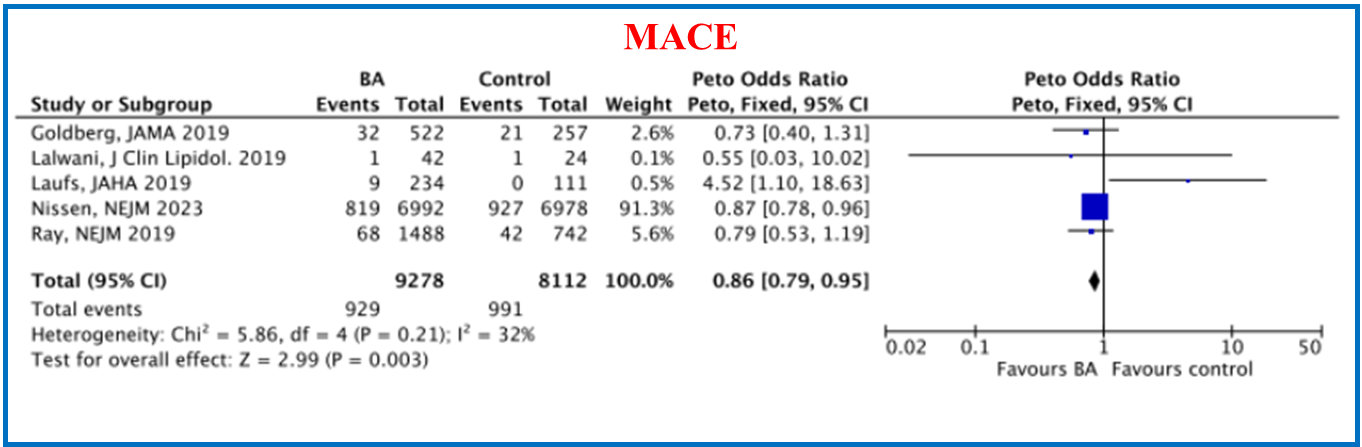


Legend as in Additional file 1: Figure S2.

**Additional file 1: Figure S6.** Efficacy of BA compared to control for percentage reduction at 12 weeks of LDL-cholesterol, total cholesterol and non-HDL cholesterol, apolipoprotein B, high-sensitivity C reactive protein (hs-CRP).


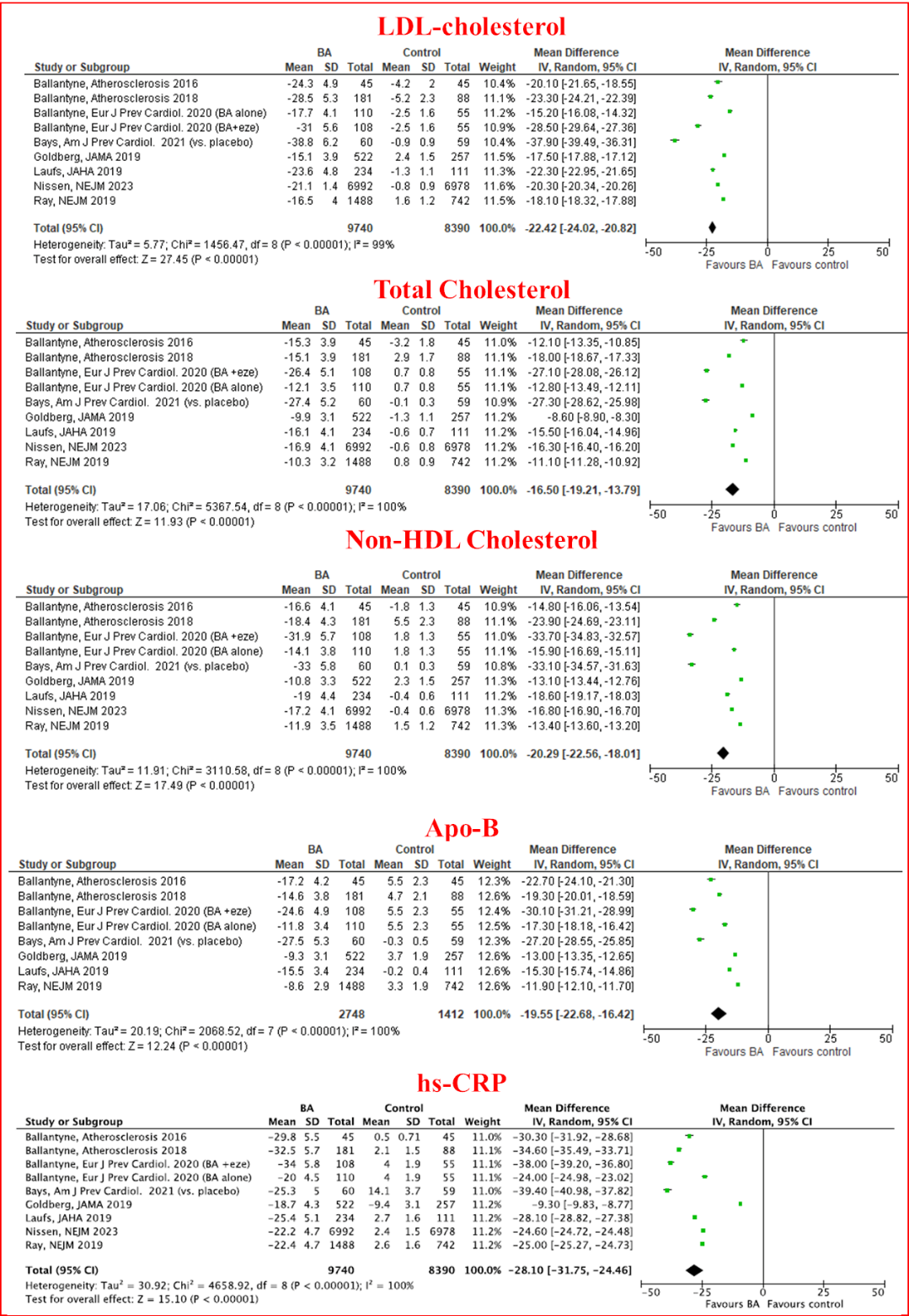


Legend as in figure 1.

**Additional file 1: Figure S7.** Efficacy of BA on laboratory endpoints at latest available follow-up


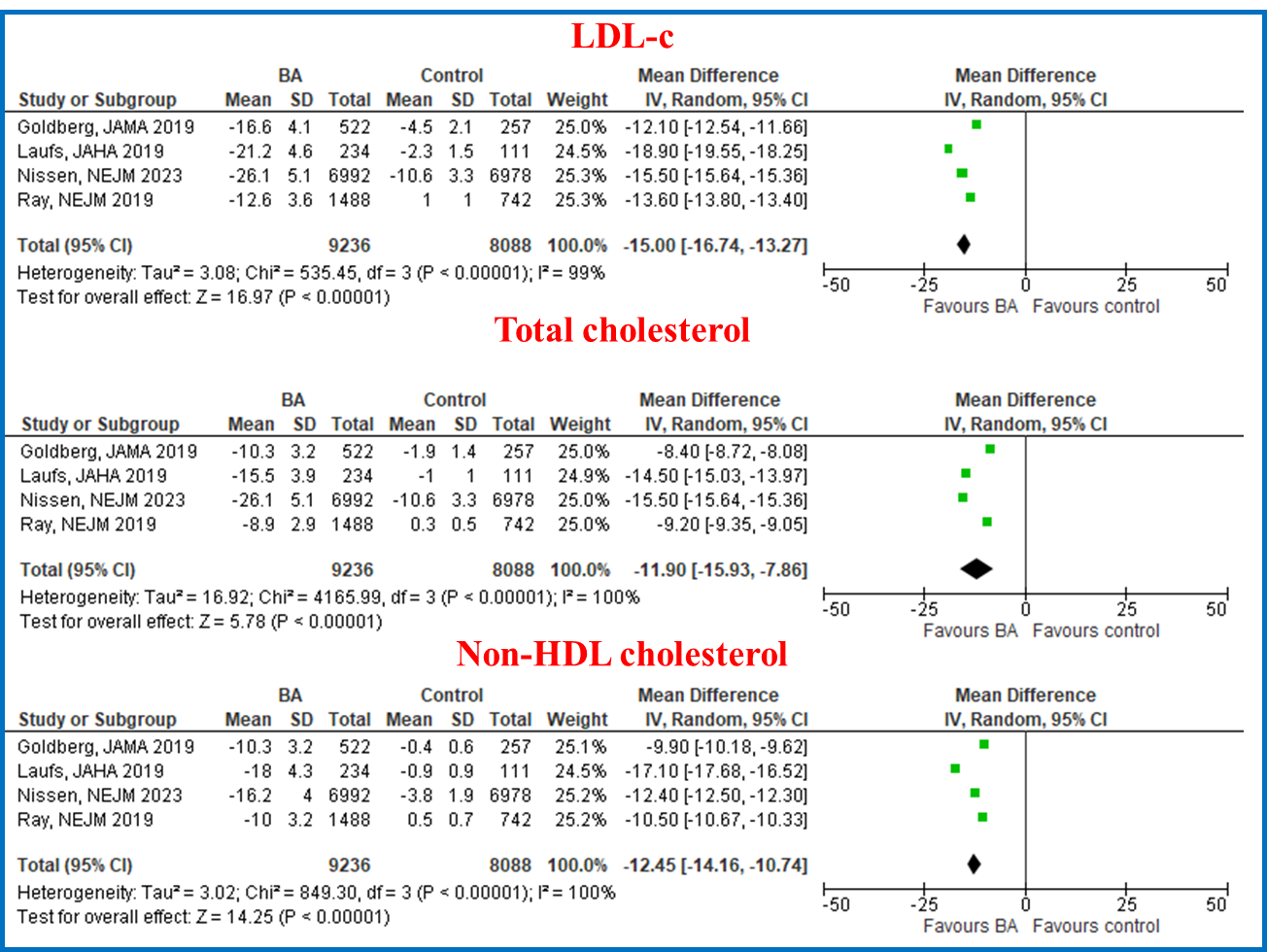


Legend as in Additional file 1: Figure S2. Data are observed over a median FU of 52 weeks (IQR, 45-79.5)

**Additional file 1: Figure S8.** Efficacy of BA on laboratory endpoints at latest available follow-up: effect on Apo-B and hs-PCR


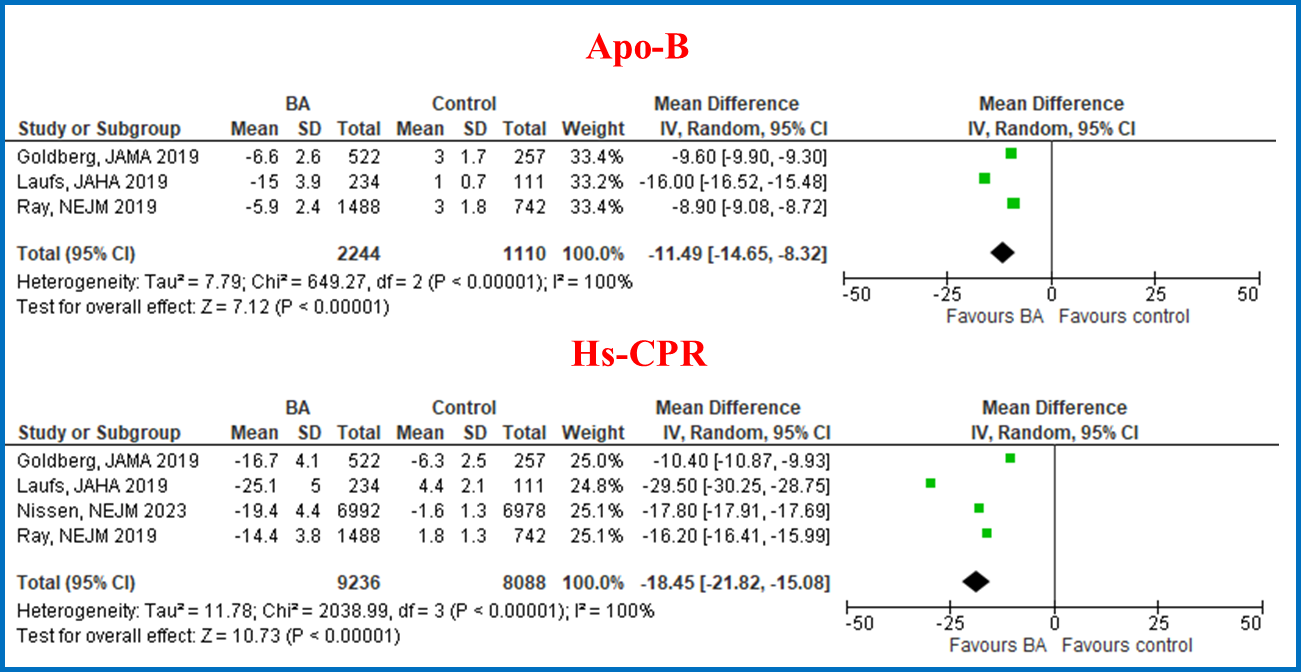


Legend as in Additional file 1: Figure S2. median observation period 52 weeks (IQR 45-79.5) for hs-CPR and 52 weeks (IQR 38-52) for ApoB.

**Additional file 1: Figure S9.** Sensitivity analysis: efficacy of BA on % reduction of LDL-c after excluding arms of BA+ ezetimibe


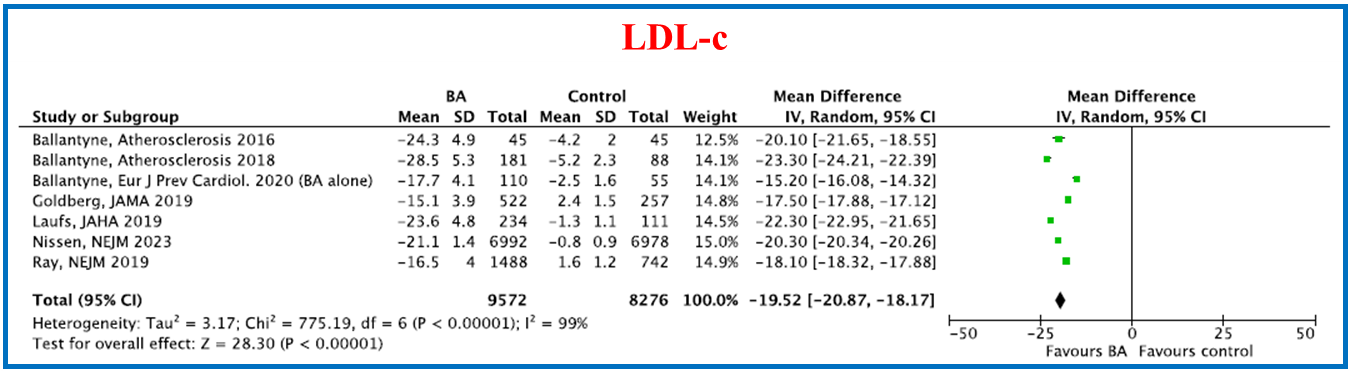


Legend as in Additional file 1: Figure S2.

**Additional file 1: Figure S10**. Efficacy of BA on LDL-C % reduction according to background medical history and trials’ inclusion criteria


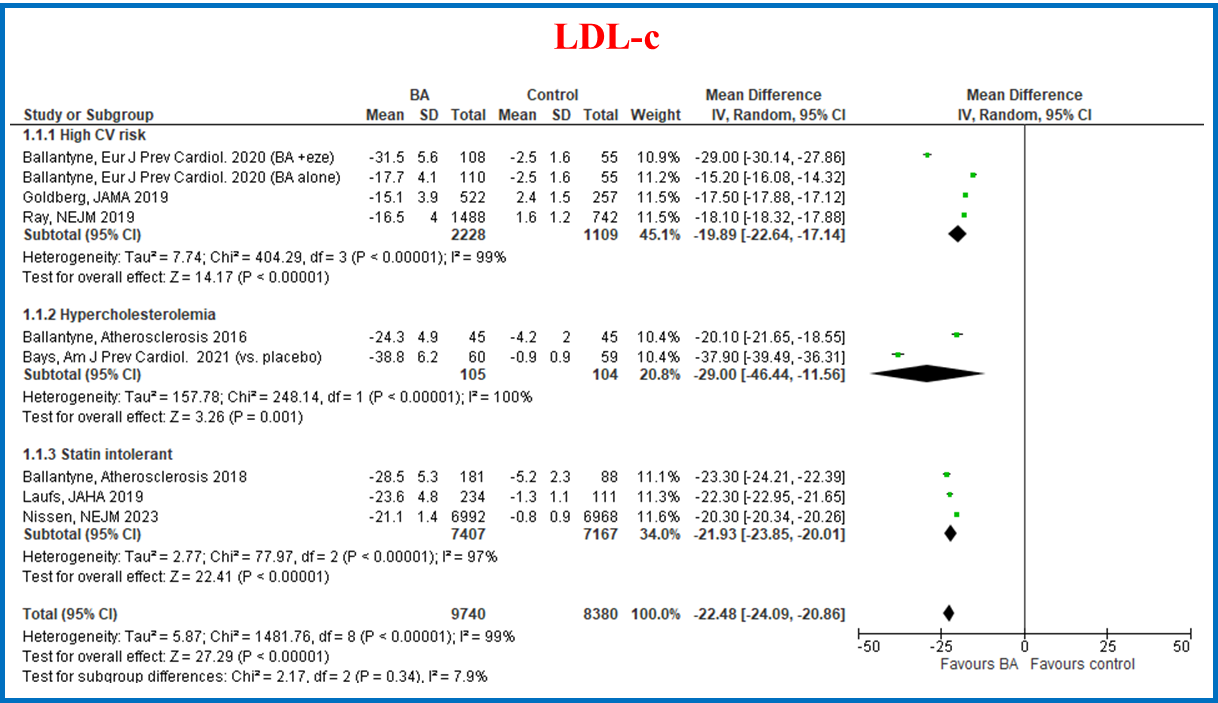


Legend as in Additional file 1: Figure S2.

**Additional file 1: Figure S11.** Efficacy of BA on total cholesterol and non HDL-cholesterol according to background medical history and trials’ inclusion criteria

**
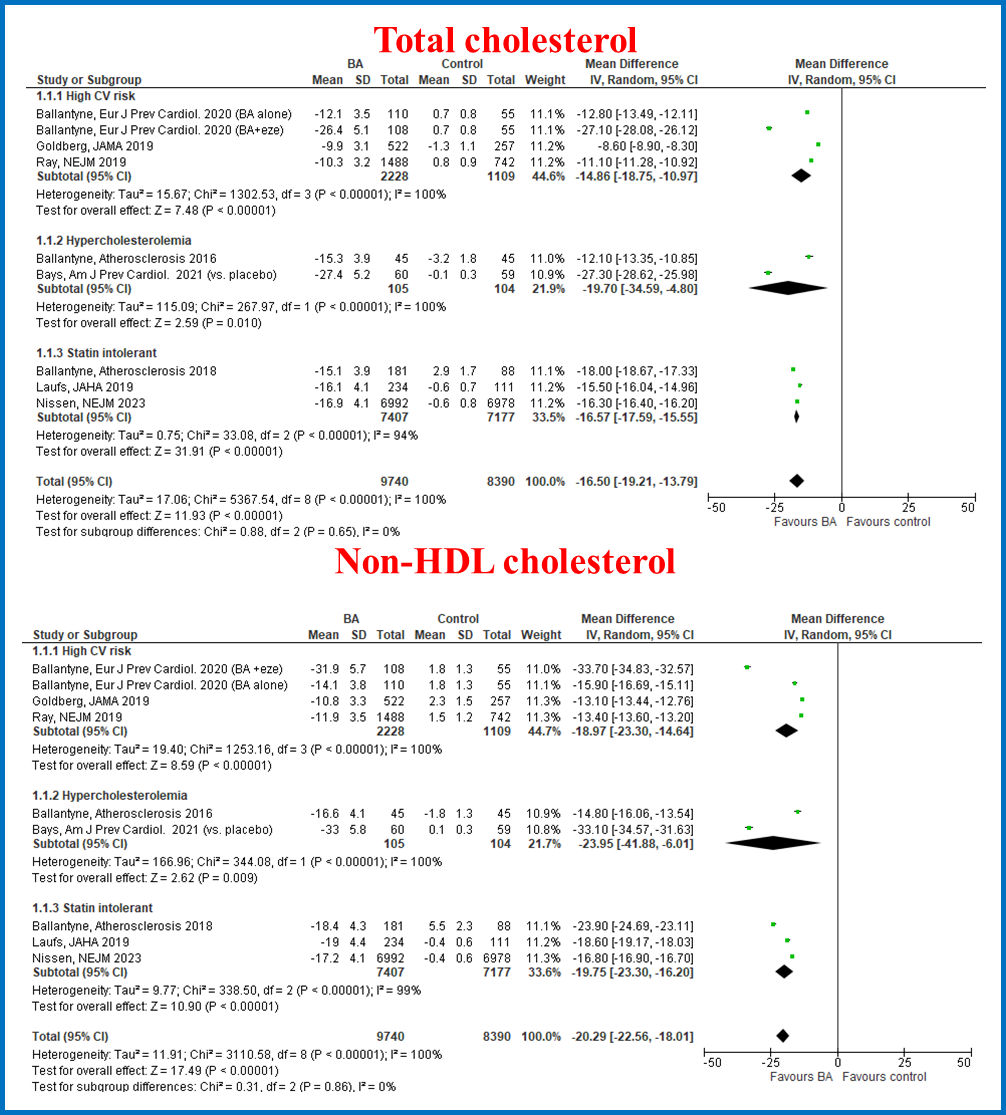
**

Legend as in Additional file 1: Figure S2

**Additional file 1: Figure S12.** Efficacy of BA on HS CPR and ApoB lipoprotein according to background medical history and trials’inclusion criteria

**
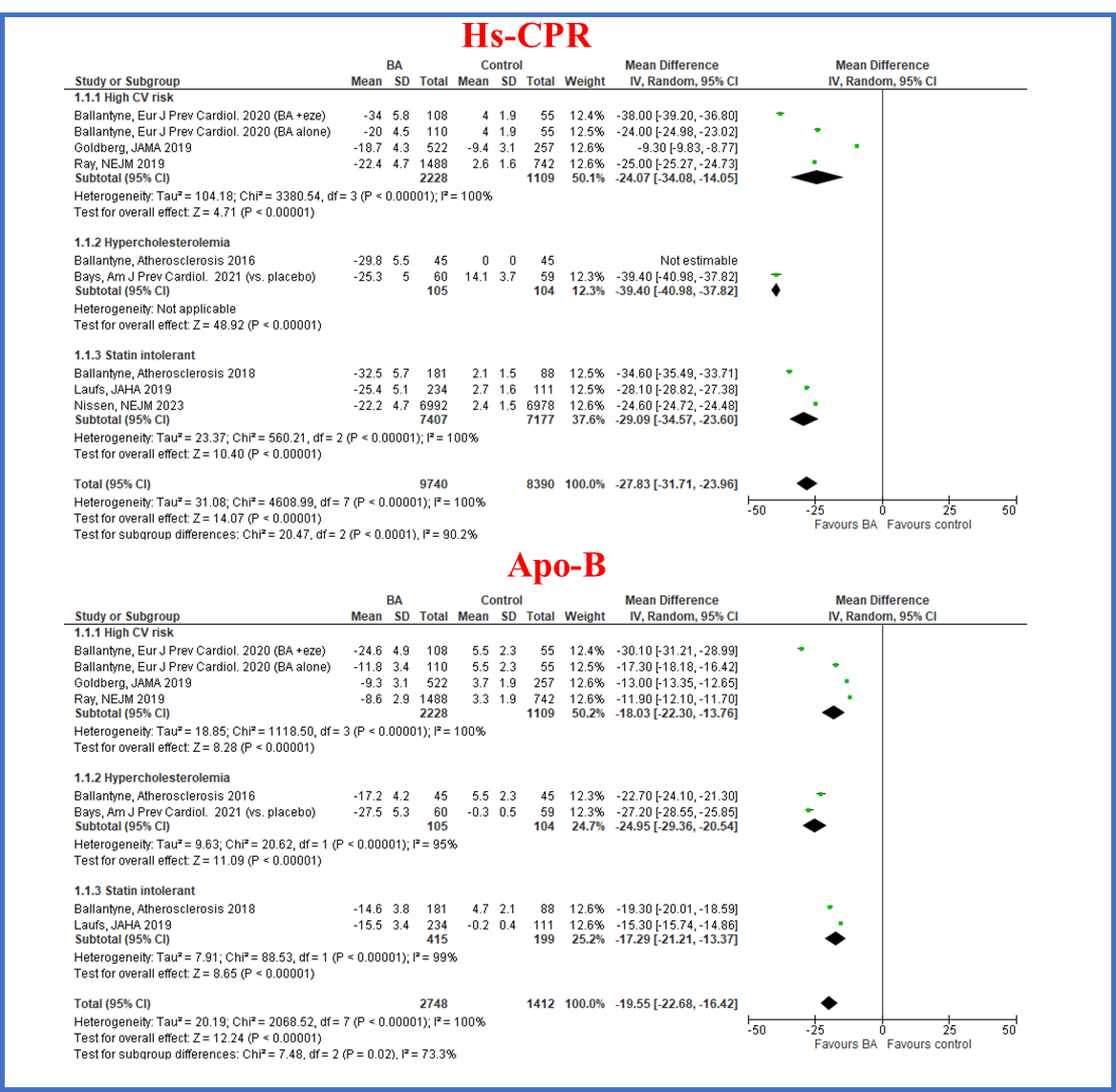
**

Legend as in Additional file 1: Figure S2

**Additional file 1: Figure S13.** Efficacy of BA on 12 weeks reduction of LDL-C according to statin background therapy


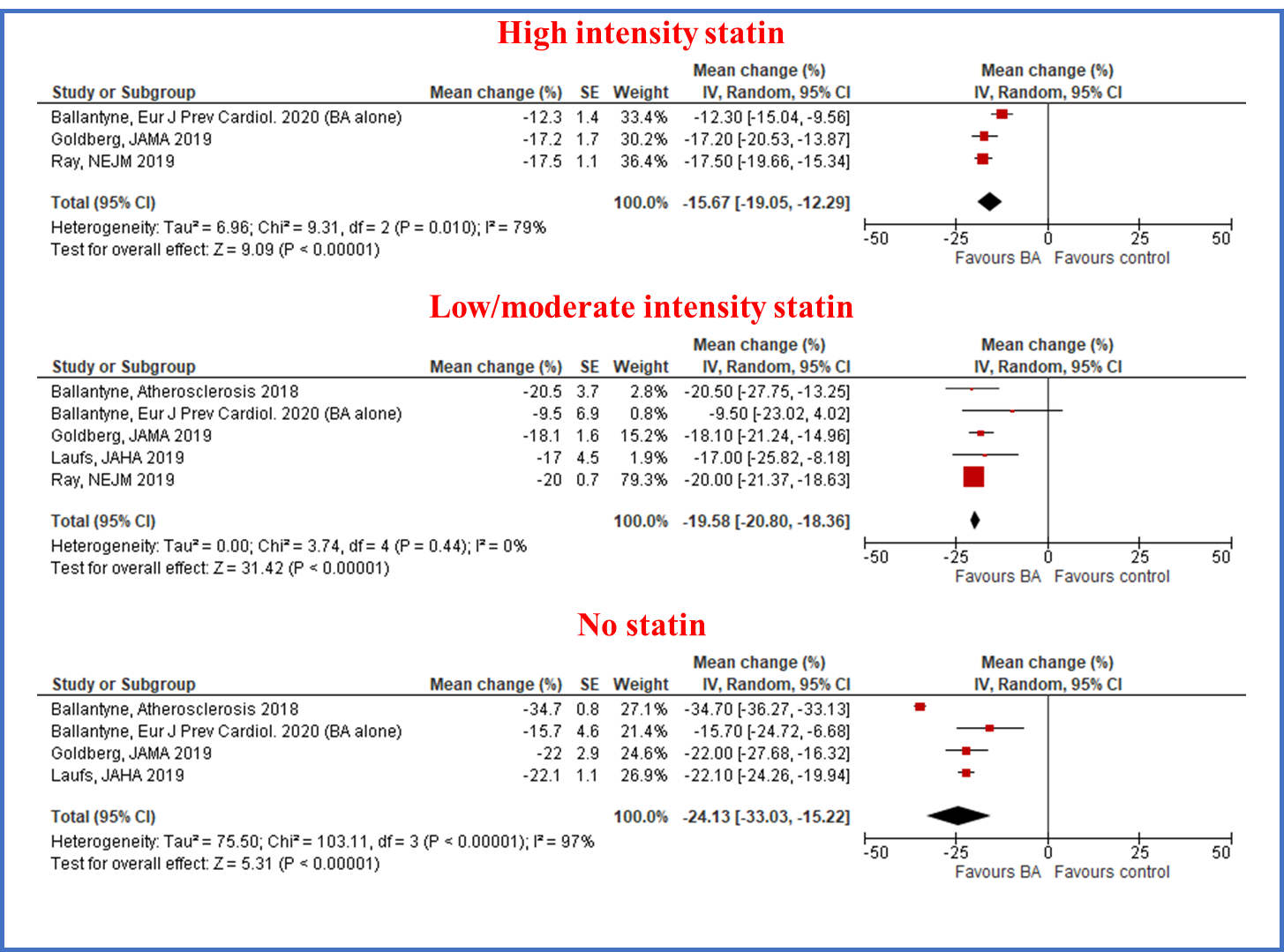


Legend as in Additional file 1: Figure S2.

**Additional file 1: Figure S14.** Efficacy of BA on LDL reduction according to background ezetimibe therapy


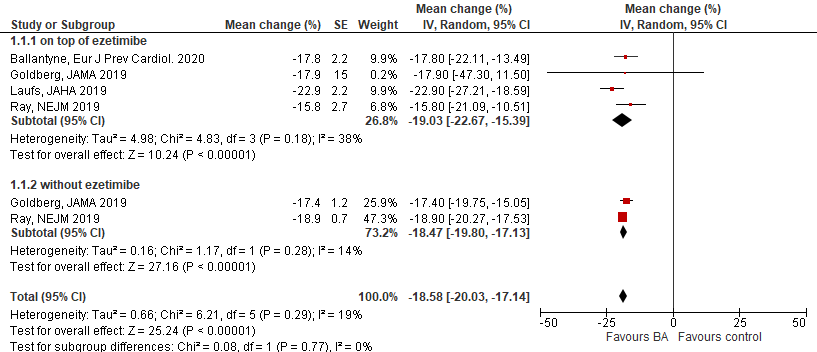


**Additional file 1: Figure S15.** Risk of any adverse event, serious adverse events and drug discontinuation due to an adverse event


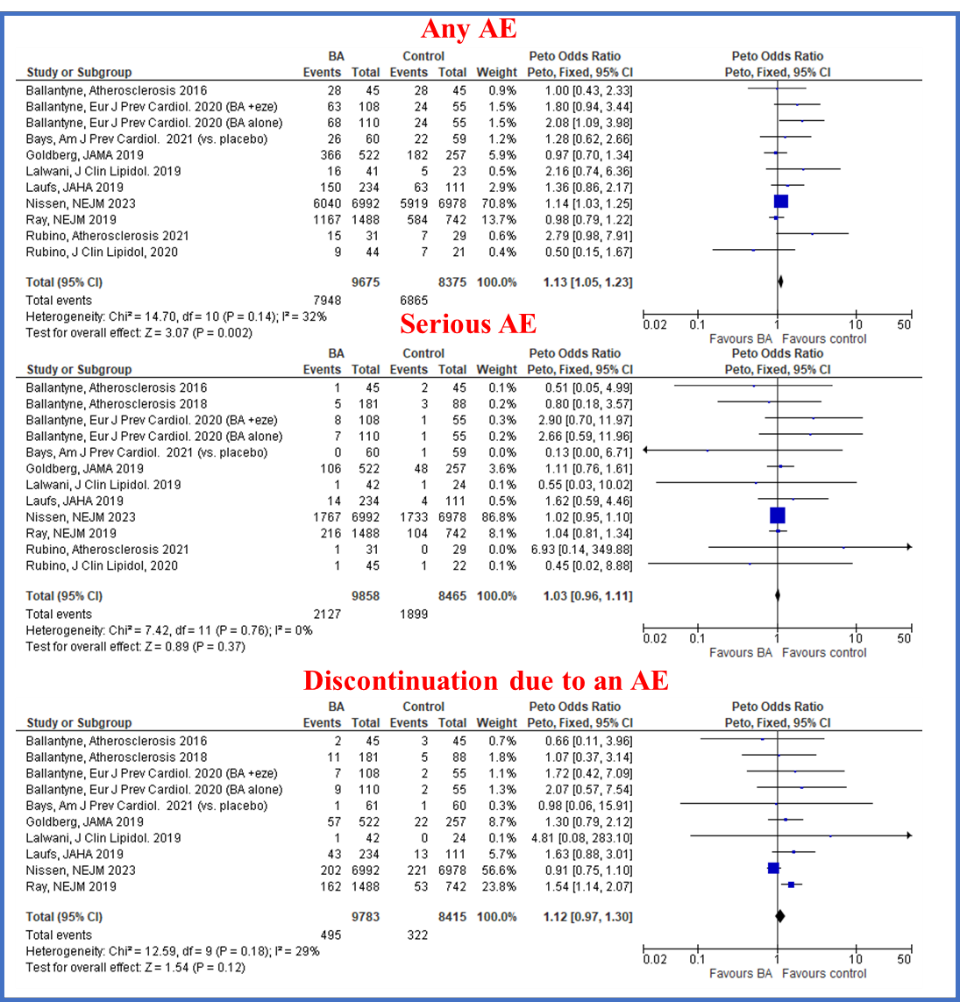


AE: adverse events. BA: bempedoic acid

**Additional file 1: Table S3.** Metaregression analysis

| **COVARIATES** |  | **MACE** | **LDL-C change** |
| --- | --- | --- | --- |
| **AGE** | *Beta* | 0.03 | 0.09 |
|  | *95% CI* | $-$0.41 to 0.48 | 0.01 to 0.16 |
|  | *P value* | 0.9 | 0.03 |
| **Male sex** | *Beta* | $-$0.009 | $-$0.002 |
|  | *95% CI* | $-$0.041 to $-$0.022 | $-$0.006 to 0.002 |
|  | *P value* | 0.6 | 0.4 |
| **Diabetes** | *Beta* | 0.001 | 0.002 |
|  | *95% CI* | $-$0.04 to 0.04 | $-$0.001 to 0.006 |
|  | *P value* | 1.0 | 0.1 |
| **Baseline cholesterol** | *Beta* | 0.003 | 0.001 |
|  | *95% CI* | $-$0.006 to 0.011 | $-$0.003 to 0.003 |
|  | *P value* | 0.5 | 1.0 |
| **Baseline LDL-c** | *Beta* | 0.004 | 0.02 |
|  | *95% CI* | $-$0.007 to 0.01 | $-$0.51 to 0.38 |
|  | *P value* | 0.5 | 0.8 |

MACE: major adverse cardiovascular events; CI: confidence intervals; LDL-C: low density lipoprotein cholesterol.

**Additional file 1: Figure S16.** Metaregression analysis. Impact of age on the risk of MACE


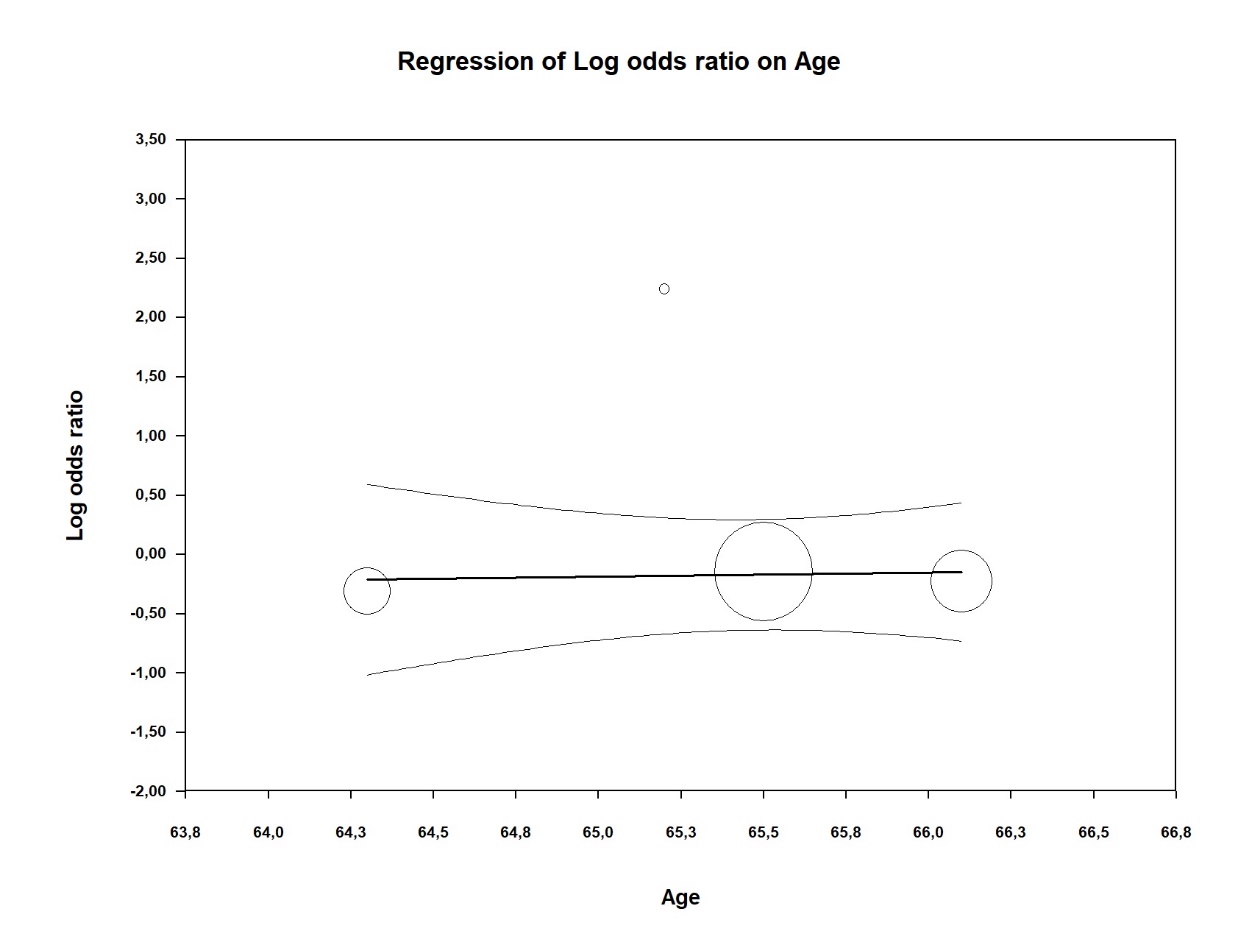


**Additional file 1: Figure S17.** Metaregression analysis. Impact of male on the risk of MACE

**
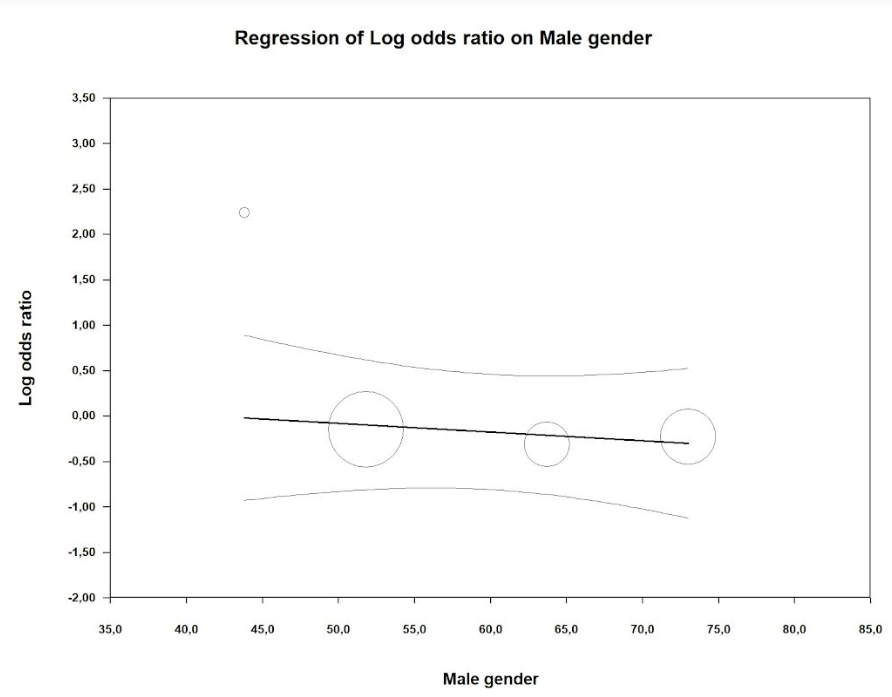
**

**Additional file 1: Figure S18.** Metaregression analysis. Impact of baseline LDL-C on the risk of MACE

**
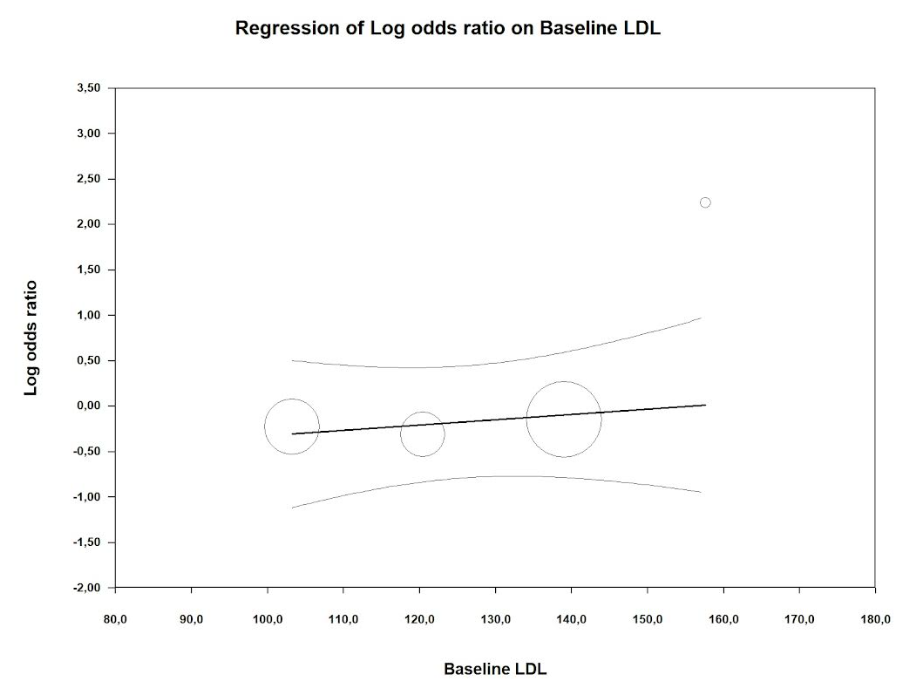
**

**Additional file 1: Figure S19.** Metaregression analysis. Impact of diabetes on the risk of MACE

**
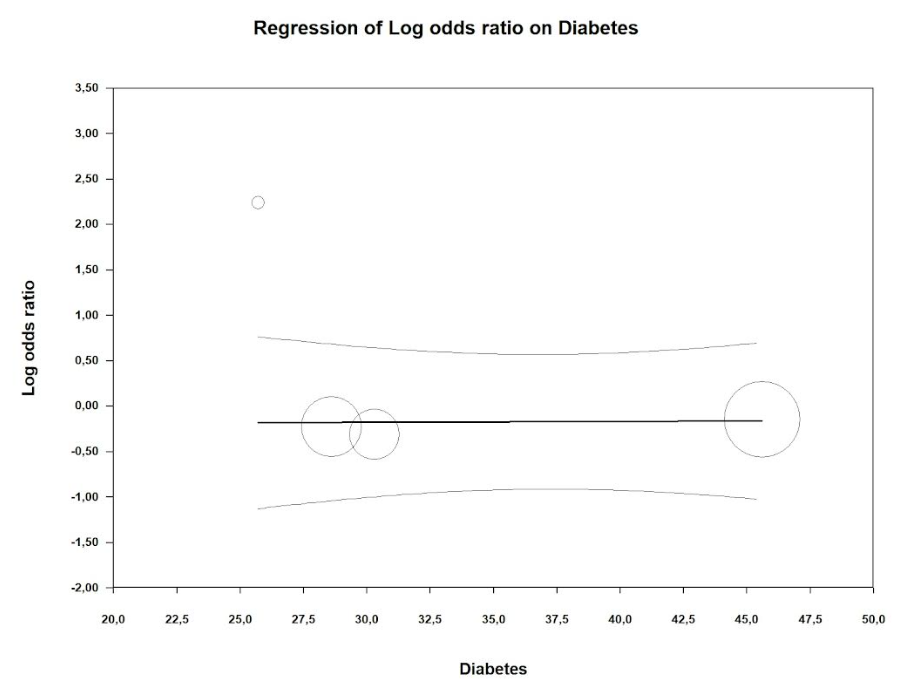
**

**Additional file 1: Figure S20.** Metaregression analysis. Impact of age on the difference in reduction of LDL-c between patients receiving bempedoic acid and control treatment group

**
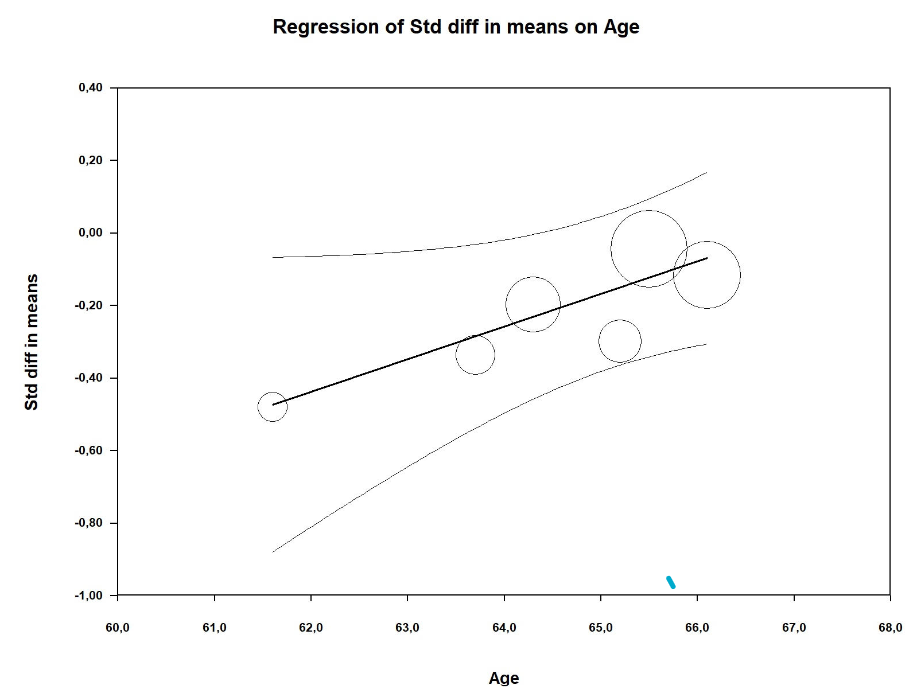
**

**Additional file 1: Figure S21.** Metaregression analysis. Impact of male gender on the difference in reduction of LDL-c between patients receiving bempedoic acid and control treatment group


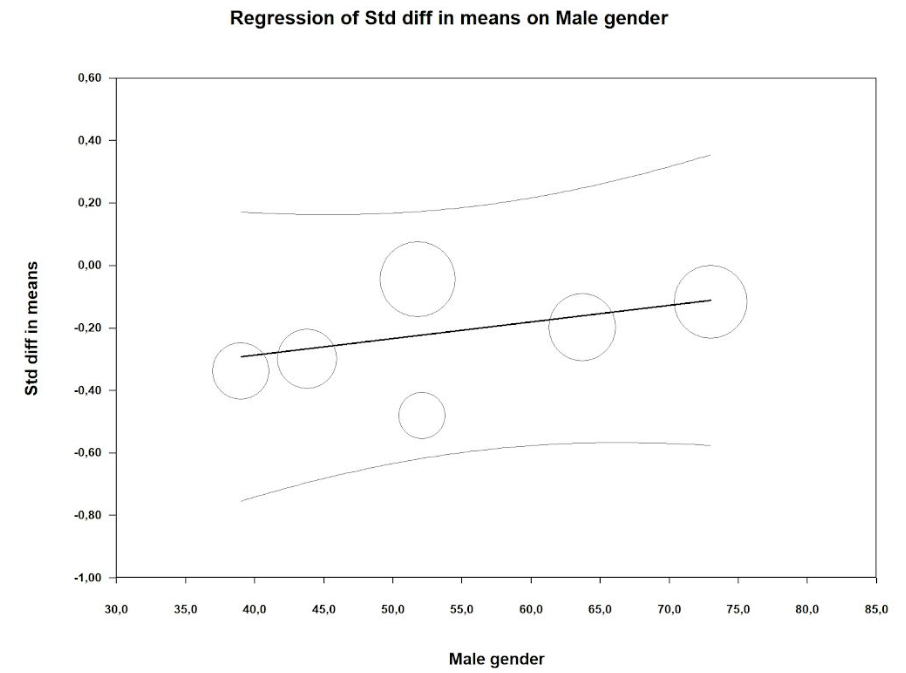


**Additional file 1: Figure S22.** Metaregression analysis. Impact of baseline LDL-c on the difference in reduction of LDL-c between patients receiving bempedoic acid and control treatment group


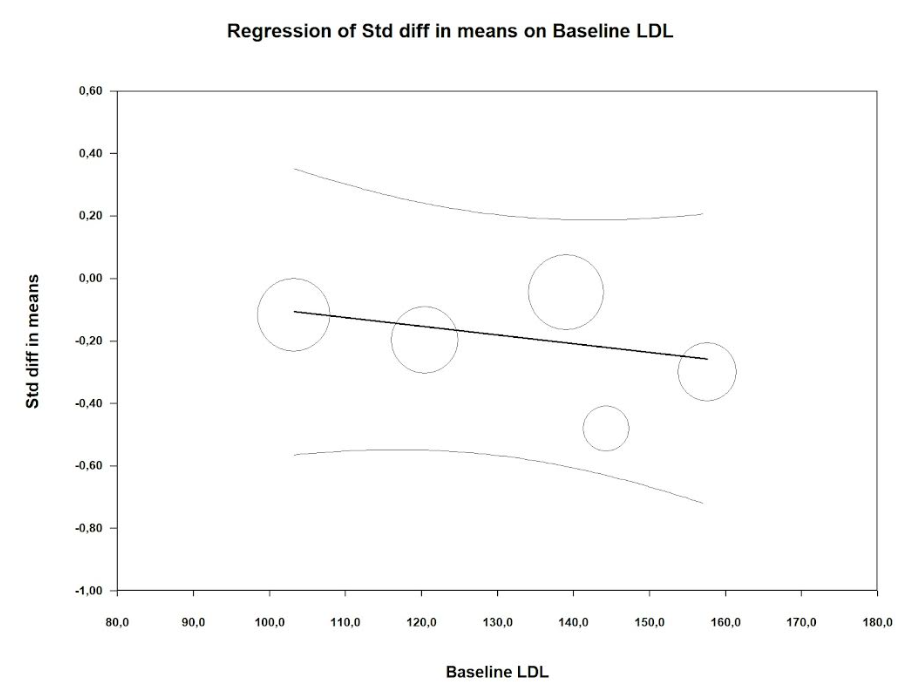


**Additional file 1: Figure S23.** Metaregression analysis. Impact of diabetes on the difference in reduction of LDL-c between patients receiving bempedoic acid and control treatment group


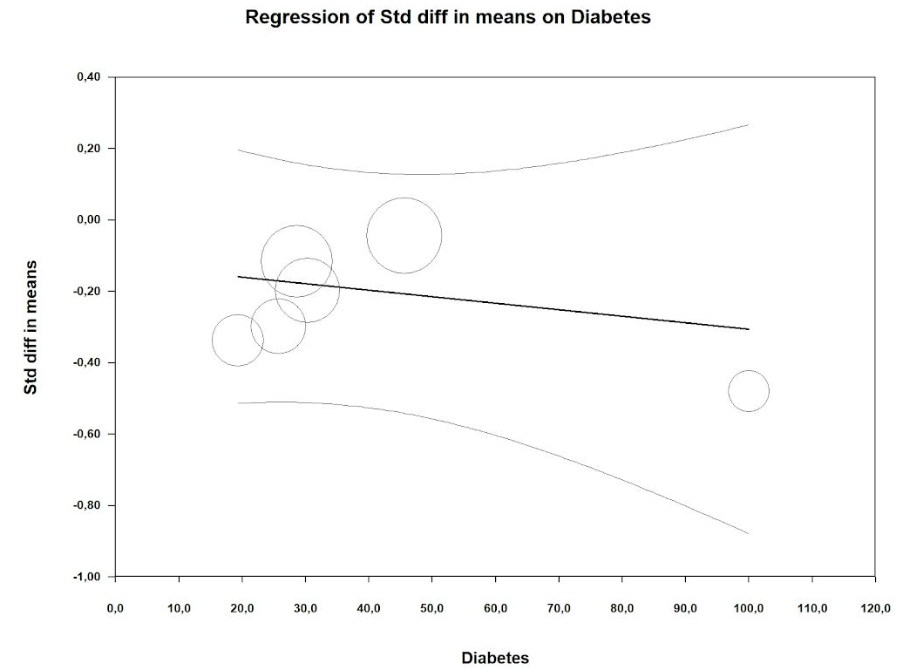

Supplement: Supplementary file 1 — Additional file 1: Figure S1. PRISMA flowchart for study selection. Table S1. Baseline features of included patients. Figure S2. Risk of Bias assessment of included trials. Figure S3. Peto Odds ratio for stroke, cardiovascular death and all-cause death.. Figure S4. Risk of MACE according to inclusion criteria and background medical history. (excel sheet provided separately). Figure S5. Sensitivity analysis for MACE (excluding trials with arms of BA and ezetimibe). Figure S6. Efficacy of BA compared to control for percentage reduction at 12 weeks of LDL-cholesterol, total cholesterol and non-HDL cholesterol, apolipoprotein B, high-sensitivity C reactive protein (hs-CRP). Figure S7. Efficacy of BA on laboratory endpoints at latest available follow-up: effect of LDL-C, total cholesterol, and non-HDL-C. Figure S8. Efficacy of BA on laboratory endpoints at latest available follow-up: effect on Apo-B and hs-PCR. Figure S9. Sensitivity analysis: efficacy of BA on % reduction of LDL-c after excluding arms of BA + ezetimibe. Figure S10. Efficacy of BA on LDL-C % reduction according to background medical history and trials’ inclusion criteria. Figure S11. Efficacy of BA on total cholesterol and non HDL-cholesterol according to background medical history and trials’ inclusion criteria. Figure S12. efficacy of BA on HS CPR and ApoB lipoprotein according to background medical history and trials’ inclusion criteria. Figure S13. Efficacy of BA on LDL reduction according to statin background therapy. Figure S14. Efficacy of BA on LDL reduction according to background ezetimibe therapy. Figure S15. Risk of any adverse event, serious adverse events and drug discontinuation due to an adverse event. Table S3. Metaregression analysis. Figure S16. Metaregression analysis. Impact of age on the risk of MACE. Figure S17. Metaregression analysis. Impact of male gender on the risk of MACE. Figure S18. Metaregression analysis. Impact of baseline LDL-C on the risk of MACE. Figure S19. Metaregre [file 12933_2023_2022_MOESM1_ESM.docx]
