# Supplementary material for: Safety and efficacy of bempedoic acid: a systematic review and meta-analysis of randomised controlled trials
Source: Cardiovasc Diabetol. 2023 Nov 28;22:324. doi: 10.1186/s12933-023-02022-z (PMC10685600; doi:10.1186/s12933-023-02022-z)
Supplement: Supplementary file 2 — Additional file 2: Table S2. Endpoint definition. MACE: major adverse cardiovascular events; AE: adverse event, TEAE: treatment-emergent adverse event, AESI: adverse events of special interest. [file 12933_2023_2022_MOESM2_ESM.docx]

**Additional file 2: Table S2.** Endpoint definition. MACE: major adverse cardiovascular events; AE: adverse event, TEAE: treatment-emergent adverse event, AESI: adverse events of special interest.

| First Author, year (Study acronym) | | MACE | | non MACE events | | Muscular disorders | | New Onset Type 2 Diabetes Mellitus | | Neurocognitive disorders | | Adverse Events | | Treatment emergent AEs | |
| --- | --- | --- | --- | --- | --- | --- | --- | --- | --- | --- | --- | --- | --- | --- | --- |
| Ray 2019,  (CLEAR HARMONY) | | CV death; non-fatal myocardial infarction; non-fatal stroke; hospitalization for unstable angina; | |  | | myalgia, muscle spasms, pain in extremity, muscular weakness (system organ class: musculoskeletal and connective tissue disorders), and creatine kinase >5 ULN (repeated and confirmed) | |  | | memory impairment, amnesia, and cognitive disorder; confusional state and disorientation | |  | |  | |
| Ballantyne 2019 | |  | |  | | muscular weakness, muscle necrosis, muscle spasms, myalgia, myoglobin blood increased, myoglobin blood present, myoglobin urine present, myoglobinemia, myoglobinuria, myopathy, myopathy toxic, necrotizing myositis, pain in extremity, and rhabdomyolysis. | |  | |  | | metabolic acidosis, hepatic safety, muscular safety, new-onset diabetes/hyperglycemia, renal safety, cardiovascular events and neurocognitive/neurological events | |  | |
| Goldberg 2019 (CLEAR WISDOM) | | cardiovascular death, myocardial infarction, nonfatal stroke, hospitalization for unstable angina, and coronary revascularization | |  | | Myalgia, muscle spasm, pain in extremities, muscular weakness, creatine kinase > 5 ULN | |  | |  | | hepatic events, muscle-related events, metabolic acidosis, hypoglycemia, new-onset or worsening diabetes, hyperuricemia, gout, renal events, and neurocognitive disorders | |  | |
| Ballantyne 2016 | |  | |  | |  | |  | |  | | AE’s coded using the Medical  Dictionary for Regulatory Activities version 16.1 | |  | |
| Laufs 2019 | | cardiovascular death,  nonfatal myocardial infarction, nonfatal stroke, hospitalization  for unstable angina, coronary revascularization | |  | | muscular  weakness, muscle necrosis, muscle spasms, myalgia, necrotizing myositis, pain in extremity, and  rhabdomyolysis. | |  | | cognitive decline, depression | |  | |  | |
| Lalwani 2019 | |  | |  | |  | |  | |  | |  | | AEs that began or worsened after the first dose of bempedoic acid or placebo. | |
| Ballantyne 2018  (CLEAR TRANQUILLITY) | cardiovascular death, nonfatal myocardial infarction, nonfatal stroke, hospitalization for unstable angina, coronary revascularization | | non-cardiovascular death, non-coronary arterial revascularization, hospitalization for heart failure | | muscle spasms, myalgia, muscular weakness, myoglobin blood increased, myoglobin blood present, myoglobin urine present, myoglobinemia, myoglinuria, myopathy, myopathy toxic, muscle necrosis, necrotizing myositis, pain in extremity, rhabdomyolysis | |  | |  | | reversible hypoglycemia, metabolic acidosis, new onset of diabetes.  Serious AE:  1) results in death  2) Is life threatening  3) Requires in-patient hospitalization or prolongation of existing hospitalization 4) Results in persistent or significant disability/incapacity, or substantial disruption of the ability to conduct normal life functions  4) Is a congenital anomaly/birth defect  5) An important medical event | |  | |  |
| Bays 2021 | |  | |  | |  | |  | |  | | AESI: adverse event of special interest: prespecified based on potential or theoretical risks of bempedoic acid or other lipid-lowering therapies and included preferred terms related to [metabolic acidosis](https://www.sciencedirect.com/topics/medicine-and-dentistry/metabolic-acidosis); hepatic, muscular, renal, cardiovascular, and neurocognitive/neurologic events; and new-onset diabetes mellitus or hyperglycemia. AESIs coded according to Medical Dictionary of Regulatory Activities (MedDRA), version 21.0. | | TEAEs: adverse events that began or worsened in severity after the first dose of double-blind study drug until 30 days after the last dose. TEAEs coded according to Medical Dictionary of Regulatory Activities (MedDRA), version 20.1 | |
| Rubino 2020 | |  | |  | |  | |  | |  | | AEs coded using the Medical Dictionary for Regulatory Activities (MedDRA) Version 19.1 or later | |  | |
| Rubino 2021 | |  | |  | |  | |  | |  | | All AEs were coded by system organ class and preferred term using the Medical Dictionary for Regulatory Affairs, version 20.1  All reports of AEs were collected from the time of informed consent until 30 days after the last dose of the IMP. | | Treatment-emergent AEs (TEAEs) were defined as AEs that began or worsened after administration of the first dose of the investigational medical product (IMP) | |
| Nissen 2023 | | death from cardiovascular causes, nonfatal myocardial infarction, nonfatal stroke, coronary revascularization | |  | | Muscular weakness, muscle necrosis, muscle spasms, myalgia, myositis, myoglobin blood increased, myoglobin blood present, myoglobin urine, myoglobin urine present, myoglobinaemia, myoglobinuria, myopathy, myopathy toxic, necrotising myositis, pain in extremity, rhabdomyolysis, blood creatine phosphokinase increased, musculoskeletal discomfort, red blood cells urine positive, muscle fatigue, muscle tightness. single incidence, and repeated and confirmed incidence1 of: CK >5 × ULN CK >10 × ULN | | one or more of the following criteria:  1) Fasting plasma glucose ≥126 mg/dL (7.0 mmol/L);  2) two-hour post-prandial glucose ≥200 mg/dL (11.1 mmol/L) during an oral glucose tolerance test as defined in the ADA guidelines; 3) HbA1C measurement ≥6.5% (48 mmol/mol); 4) in patient with classic symptoms of hyperglycemia or hyperglycemic crisis, a random plasma glucose ≥200 mg/dL (11.1 mmol/L)  5) unequivocal hyperglycemia symptoms | | Cognitive disorder, confusional state, disorientation, memory impairment, mental status changes | | Tendon Rupture confirmed by diagnostic findings or 1 symptom and 1 PE finding that are consistent with the rupture type.  AEs: any untoward medical occurrence in a clinical investigation patient administered a pharmaceutical product, including control, and which does not necessarily have a causal relationship with treatment. | | AEs that begin or worsen after the first dose of double-blind IMP and through the end of the study including the follow-up period. | |
